# Fungal and Toxin Contaminants in Cereal Grains and Flours: Systematic Review and Meta-Analysis

**DOI:** 10.3390/foods12234328

**Published:** 2023-11-29

**Authors:** Christodoulos Deligeorgakis, Christopher Magro, Adriana Skendi, Haileeyesus Habtegebriel Gebrehiwot, Vasilis Valdramidis, Maria Papageorgiou

**Affiliations:** 1 Department of Food Science and Technology, International Hellenic University, P.O. Box 141, GR-57400 Thessaloniki, Greece; deligeorgakis@food.ihu.gr; 2 Department of Food Sciences and Nutrition, Faculty of Health Sciences, University of Malta, MSD 2080 Msida, Malta; christopher.magro.17@um.edu.mt (C.M.); haileeyesus.gebrehiwot@um.edu.mt (H.H.G.); vasilis.valdramidis@chem.uoa.gr (V.V.); 3 Laboratory of Food Chemistry, Department of Chemistry, National and Kapodistrian University of Athens, Zografou, GR-15771 Athens, Greece

**Keywords:** cereals, wheat flour, toxigenic fungi, mycotoxins, meta-analysis, HPLC-MS/MS, deoxynivalenol, aflatoxins, *Fusarium*

## Abstract

Cereal grains serve as the cornerstone of global nutrition, providing a significant portion of humanity’s caloric requirements. However, the presence of fungal genera, such *Fusarium*, *Penicillium*, *Aspergillus*, and *Alternaria*, known for their mycotoxin-producing abilities, presents a significant threat to human health due to the adverse effects of these toxins. The primary objective of this study was to identify the predominant fungal contaminants in cereal grains utilized in breadmaking, as well as in flour and bread. Moreover, a systematic review, including meta-analysis, was conducted on the occurrence and levels of mycotoxins in wheat flour from the years 2013 to 2023. The genera most frequently reported were *Fusarium*, followed by *Penicillium*, *Aspergillus*, and *Alternaria*. Among the published reports, the majority focused on the analysis of Deoxynivalenol (DON), which garnered twice as many reports compared to those focusing on Aflatoxins, Zearalenone, and Ochratoxin A. The concentration of these toxins, in most cases determined by HPLC-MS/MS or HPLC coupled with a fluorescence detector (FLD), was occasionally observed to exceed the maximum limits established by national and/or international authorities. The prevalence of mycotoxins in flour samples from the European Union (EU) and China, as well as in foods intended for infants, exhibited a significant reduction compared to other commercial flours assessed by a meta-analysis investigation.

## 1. Introduction

For over ten thousand years, cereals have been used by humans as a staple crop, playing a crucial role in their diets and providing essential energy in the form of carbohydrates, proteins, lipids, and vitamins [1,2]. Cereals, such as rice (*Oryza sativa*), wheat (*Triticum aestivum* L.), and maize (*Zea mays* L.), are of prime importance for human food and are grown in many areas around the world [3]. Notwithstanding, other crops, such as barley (*Hordeum vulgare* L.), durum wheat (*Triticum durum Desf.*), sorghum (*Sorghum bicolor* L.), oat (*Avena sativa* L.), rye (*Secale cereale* L.), and millet (*Panicum milliaceum* L.), also make up the significant portion of the human diet.

Cereals face major challenges both from biotic and abiotic stressors during cultivation, such as climatic changes, but also from fungal infection. Infection may happen by fungi, which can ultimately degrade the quality of the product during pre- and post-harvest as well during storage. Inappropriate storage remains the major threat that should not be ignored [4]. It is estimated that within the next two or three decades, the yield would most probably decrease by more than 25%, and given the exponential rise of the human population, production will hardly satisfy world demand [5,6]. Moreover, the average yield losses due to fungal contamination in cereals are estimated to be around 15–20%, the maximum extending up to 50% [7]. In 2019, 22% of wheat yield losses were entirely due to fungal diseases [8].

Grains are the basis for the manufacturing of a wide range of goods that are prepared with flour or meals, such as baked products (breads, cookies, and cakes), breakfast cereals, pasta, soups, and gravies, while they can also be consumed as wholegrain or can be fermented to produce beverages [9]. About 50% of the world’s calories are obtained from cereal grain consumption [3], while wheat bread alone provides more nutrients to the world population than any other food source [10,11].

Those challenges related to fungal contamination not only spoil and ruin the quality of the produce but also can cause adverse effects on health due to their ability to produce a range of metabolites known as mycotoxins [4]. Mycotoxins are estimated to be present in 25% of the world’s harvested crops leading to five billion dollars in losses annually in the United States and Canada only [12,13]. Overall, over 400 compounds are defined as mycotoxins, while 30 of them are given more importance since they are deleterious to human and animal health [14].

Mycotoxins originate from the Greek word “μύκητας-mykitas”, which means fungus, and the Latin word “toxicum”, meaning poison. They are low molecular weight compounds, naturally present in cereals, which also act as secondary metabolites produced mainly by mycelial structures of filamentous fungi that do not exhibit any biochemical meaning to fungus growth and development [15]. Greeks and Romans were probably aware of illnesses caused by fungi, while documents from the Middle Ages reported various sicknesses from mycotoxins [16]. These diseases affect all aspects of human and animal health. In particular, they provoke acute and chronic diseases due to being carcinogenic, mutagenic, teratogenic, estrogenic, hemorrhagic, immunotoxic, nephrotoxic, hepatotoxic, dermatoxic, and neurotoxic [17]. Contamination of cereals by mycotoxins can occur during cultivation in the field, processing, storage, and/or during transportation. Their consumption can either be directly through the consumption of contaminated food or indirectly through the consumption of animal products, such as meat, milk, and eggs from animals fed with mycotoxin-contaminated feed. Since most mycotoxins exhibit chemical and thermal stability during food processing [18,19], a general instruction for the minimization of the risk of mycotoxins advice the application of GAPs (Good Agricultural Practices) and HACCP (Hazard Analysis Critical Control Points) at pre- and post-harvest [20].

Regulations regarding mycotoxins are stipulated based on the scientific opinions of authoritative bodies, such as the Food Agriculture Organization (FAO), the World Health Organization (WHO), the Joint Expert Committee on Food Additives of the United Nations (JECFA), the European Food Safety Authority (EFSA), that have replaced the pre-existing national regulations [21]. Currently, they are jointly synchronized into a norm followed by different members of economic communities (e.g., EU (European Union), MERCOSUR (Mercado Cómun del Sur), Association of Southeast Asian (ASEAN), Australia and New Zealand, and others). However, up to now, there are countries around the world that still lack regulatory limits, or in the case that there are any limits established, they are applied exclusively to international trade [22]. Classification of mycotoxins based on their carcinogenic potency is established by the International Agency for Research on Cancer (IARC), while the Joint FAO/WHO Expert Committee on Food Additives (JECFA) has established a tolerable daily/weekly intake (TDI/TWI) based on the consumption of food over a lifetime without risk of adverse health effects [23].

The goal of this review is to present the most common fungal contaminants found in cereal grains and cereal flours, the activity of which is responsible for the generation of mycotoxin(s). It also focuses on the occurrence and concentration of mycotoxins in wheat flours reported worldwide between 2013 and 2023, complemented by a comprehensive meta-analysis.

## 2. Materials and Methods

### 2.1. Search Strategy

A comprehensive search was conducted to gather sources studying mycotoxins, including aflatoxins, fumonisins, trichothecenes, zearalenone, and novel mycotoxins in wheat flour. The systematic review focused on published articles, excluding reviews, spanning the years 2013 to 2023, i.e., focusing on the last decade. Databases, such as Web of Science (https://mjl.clarivate.com/search-results, accessed on 10 April 2023), Scopus (https://www.scopus.com/search/form.uri?display=basic&zone=header&origin=#basic, accessed on 10 April 2023), and Elsevier (https://www.sciencedirect.com/search, accessed on 10 April 2023), were utilized to collect the studies.

The search strategy employed keywords: [wheat flour] AND (Mycotoxins OR Aflatoxins OR Fumonisins OR Trichothecenes OR Zearalenone OR Novel Mycotoxins) AND (Incidence OR Occurrence OR Prevalence OR Contamination). In Web of Science and Elsevier, the search term was: ((“wheat flour”) AND (Mycotoxins OR Aflatoxins OR Fumonisins OR Trichothecenes OR Zearalenone OR “Novel Mycotoxins”) AND (Incidence OR Occurrence OR Prevalence OR Contamination)). In Scopus, the term was: title-abs key (“wheat flour”) AND title-abs-key (Mycotoxins) OR title-abs-key (Aflatoxins) OR title-abs-key (Fumonisins) OR title-abs-key (Zearalenone) OR title-abs-key (“Novel Mycotoxins”) AND title abs- key (Incidence) OR title-abs-key (Occurrence) OR title-abs-key (Prevalence) OR title-abs-key (Contamination).

This search yielded a total of 566 articles, and after removing 124 duplicates, the remaining 442 articles were screened based on title, keyword, and abstract. After this process, 164 articles were screened for content, resulting in the selection of 69 articles.

### 2.2. Statistical Analysis and Meta-Analysis

The data in Table 4 were categorized into three specific categories to perform the meta-analysis: the geographical area specifying the continent of origin of the samples, the type of mycotoxins, and the flour type, i.e., white flour, flour intended for infants, and whole wheat flour. The analysis was carried out using the log odds as the outcome measure. A mixed-effects model (k = 208; tau^2^ estimator: REML) was used to examine the effect of moderators (area, type of mycotoxin, and flour type) on the level of prevalence of mycotoxins. The amount of heterogeneity (i.e., τ2), was estimated using the restricted maximum-likelihood estimator [24,25]. In addition to τ2, the Q-test for heterogeneity [25,26] and the I^2^ statistic [26,27] were reported. In case any amount of heterogeneity was detected (i.e., τ2 > 0, regardless of the results of the Q-test), a prediction interval for the true outcomes was also provided [27,28]. Studentized residuals and Cook’s distances were used to examine whether studies may be outliers and/or influential in the context of the model [28,29]. Studies with a studentized residual larger than the 100 × (1 − 0.05/(2 × k))th percentile of a standard normal distribution were considered potential outliers (i.e., using a Bonferroni correction with two-sided α = 0.05 for k studies included in the meta-analysis). Studies with a Cook’s distance larger than the median plus six times the interquartile range of the Cook’s distances were considered to be influential. The rank correlation test [29,30] and the regression test [30,31], using the standard error of the observed outcomes as a predictor, were used to check for funnel plot asymmetry. The analysis was carried out using R (version 4.2.1) [31,32] and the metafor package (version 3.8.1) [32,33]. The logit transformed proportion (PLO) used in this analysis is expressed as:

(1) $$\log\left(\frac{x_{i}}{n_{i}-x_{i}}\right)$$

where, $x_{i}$ is the number of positive samples whereas $n_{i}$ is the number of total samples included in the study.

## 3. Fungal Contamination in Grains Used for Breadmaking

### 3.1. Main Fungal Contaminants in Cereal Grains Used in Breadmaking

It is expected that due to global warming, conditions are ever becoming more favorable to fungal infections due to higher temperatures, humidity, moisture content, and rainfalls [7,34]. For instance, *Fusarium* spp. infections are expected to increase in their emergence in Europe [34,35].

Any cereal cultivar can have several sources of contamination from which fungi can inoculate and accumulate. Some examples are the soil, water, harvesting bins, machinery, tools, and composted manure [36]. Not to mention the wind patterns, which can further create problematic scenarios since fungal spores are ubiquitous, causing endless dispersal and contamination of cereals and their derived products [37]. The main fungal pathogens to grains that are of concern are *Fusarium* spp., *Penicillium* spp., *Aspergillus* spp., and *Alternaria* spp. [4]. They are deleterious and toxic fungi with the potential to grow in a myriad of environmental conditions and the ability to produce mycotoxins [38]. *Fusarium* spp., *Alternaria* spp., *Cladosporium* spp., and *Helmintosporium* spp. are normally found contaminating the cereals in the field, while other contaminants have a higher incidence during storage, such as *Penicillium* spp., *Aspergillus* spp., *Eurotium* spp., and *Rhizopus* spp. [39]. This makes fungal contamination unavoidable along the whole value chain [36,38].

**Figure 1:**
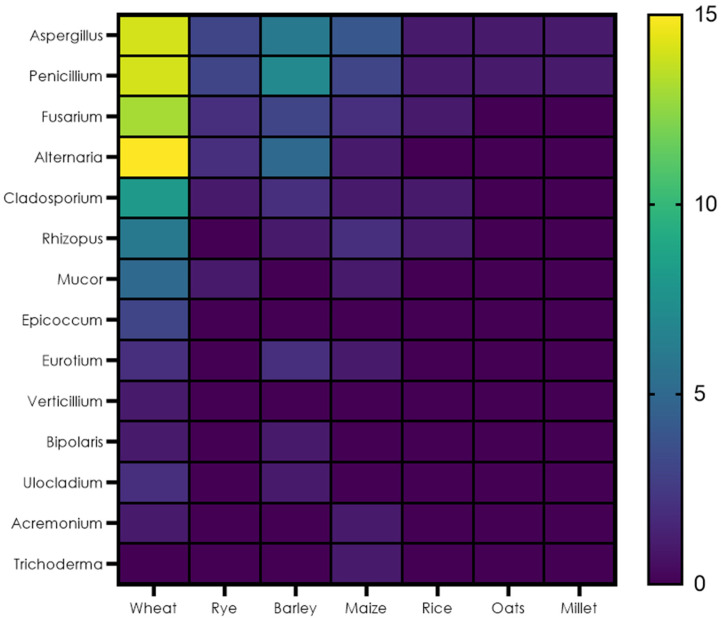
Heat map of the contamination occurring in cereals based on data presented in Table 1.

A heat map was generated as shown in Figure 1, to depict the fungal contamination data for cereals presented in Table 1. From a total of 18 studies, all performed fungal identification assessments on different cereals used in the bread industry, wheat (65%) and barley (26%) were the most common cereals reported. As for wheat, known pre- and post-harvest contaminants [40], as well as mycotoxin producing fungal genera, were the most frequently isolated, such as *Alternaria* spp. (88%), *Aspergillus* spp. (82%), *Penicillium* spp. (82%), followed by *Fusarium* spp. (76%). *Alternaria alternata*, *Aspergillus flavus*, *Penicillium citrinum*, *Penicillium expansum*, *Fusarium graminearum*, and *Fusarium avenaceum* all producing mycotoxins of public and health concerns, such as alternariol (AOH) [41], aflatoxins [42], citrinin [43], patulin [44], deoxynivalenol (DON), and zearalenone (ZEA) [45]. Regarding barley, the main fungal contaminants were similar to those of wheat but different in incidence as *Penicillium* spp. (100%) was the most abundant genera isolated, followed by *Aspergillus* spp. (86%), *Alternaria* spp. (71%), and *Fusarium* spp. (42.8%) with the latter being the least abundant fungal isolate, as seen in wheat. Rye and maize also showed a high prevalence of these fungal genera when compared to other fungal contaminants, as shown in Figure 1. *Trichoderma*, *Acremonium*, *Bipolaris*, *Ulocladium*, *Eurotium*, and *Epicoccum* are fungal contaminants that are very scarcely reported in cereals.

The most important fungal disease caused in cereals, such as wheat and barley, is *Fusarium* head blight (FHB) [46]. This disease is chiefly caused by *Fusarium graminearum* and *F. culmorum*. Both organisms are known to produce highly toxic metabolites, namely deoxynivalenol (DON) and zearalenone (ZEA). Both mycotoxins are a significant threat to human and animal health [47]. DON is known to cause vomiting, abdominal pain, fever, and headaches, while ZEA is known to affect the reproductive system, in particular, estrogen hormone, can cause hepatocarcinoma in the liver and also affect the immune system [48,49]. FHB is also troublesome since it causes the spoilage of wheat and its grains, diminishing the yield and degrading the quality of the grain, compromising the safety and security of the food [35].

Table 1 compiles the main fungal contaminants in various cereal grains, which have been reported through various studies, where the majority cause spoilage and disease to the cereal but also potentially produce mycotoxins along with genera of *Aspergillus* spp., *Penicillium* spp., *Fusarium* spp., and *Alternaria* spp. Other detected organisms are contaminants that bring about spoilage to bakery products, such as *Rhizopus* spp. and *Mucor* spp., which are primarily responsible for the black bread mold.

**Table 1:** Occurrence of fungal contaminants, load, and detection methods in cereal grains.

Cereal Type	Location	Number of Samples Tested	Fungal Species ^1^ (Frequency, %)	Fungal Load (log CFU/g unless Stated Differently) ^2^	Detection Techniques Applied	References
Wheat	Tunisia	n = 24	*Aspergillus* spp. (54.17%), *Penicillium* spp. (41.67%), *Alternaria* spp. (70.83%), *Fusarium* spp. (9.52%) *Eurotium* spp. (62.5%), *Cladosporium* spp. (29.17%), *Rhizopus* spp. (4.17%)	Not specified	Direct Plating Techniques (PDA) and DNA-Based Techniques to detect *Fusarium* and *Aspergillus* toxigenic strains	[50]
Barley	Tunisia	n = 20	*Aspergillus* spp. (70%), *Penicillium* spp. (75%), *Eurotium* spp. (65%), *Alternaria* spp. (65%), *Fusarium* spp. (25%), *Rhizopus* spp. (25%), *Cladosporium* spp. (25%)	Not specified	Direct Plating Techniques (PDA) and DNA-Based Techniques to detect Fusarium and Aspergillus toxigenic strains	[50]
Maize	Tunisia	n = 21	*Aspergillus* spp. (76.19%), *Penicillium* spp. (38.10%), *Fusarium* spp. (19.05%), *Alternaria* spp. (14.29%), *Cladosporium* spp. (20%), *Rhizopus* spp. (14.29%)	Not specified	Direct Plating Techniques (PDA) and DNA-Based Techniques to detect Fusarium and Aspergillus toxigenic strains	[50]
Wheat	Poland	n = 129	*Aspergillus* spp. (20%), *Penicillium* spp. (20%), *Alternaria* spp. (10%), *Fusarium* spp. (13%), *Mucor* spp. (11%), *Cladosporium* spp. (6%)	Mean for 129 samples = 1.43	Direct Plating Techniques (DRBC)	[2]
Wheat	Turkey	n = 90	*A. candidus*, *A. clavatus*, *A. flavus*, *A. fumigatus*, *A. niger*, *A. orhraceus*, *A. wentii*, *P. expansum*, *P. viridictum*, *P. chrysogenum*, *Cladosporium* spp., *Rhizopus* spp., *Ulocladiumspp.*, *Alternaria alternata*, *Acremonium* spp., *Mucor* spp.	Not specified	Direct Plating Techniques (MEA)	[51]
Rice	Turkey	n = 60	*A. candidus*, *A. clavatus*, *A. flavus*, *A. fumigatus*, *A. niger*, *A. versicolor*, *P. chrysogenum*, *P. citrinum*, *P. expansum*, *P. cyclopium*, *Cladosporium* spp., *Rhizopus* spp., *Fusarium* spp.	Not specified	Direct Plating Techniques (MEA)	[51]
Corn	Turkey	n = 21	*A. flavus*, *A. niger*, *A. wentii*, *P. chrysogenum*, *P. citrinum*, *P. expansum*, *P. cyclopium*, *Acremonium* spp., *Cladosporium* spp., *Eurotium* spp., *Rhizopus* spp.	Not specified	Direct Plating Techniques (MEA)	[51]
Barley	Turkey	n = 9	*A. flavus*, *A. versicolor*, *P. chrysogenum*, *P. expansum*,	Not specified	Direct Plating Techniques (MEA)	[51]
Wheat	Brazil	n = 150	*Fusarium* spp., *Alternaria* spp., *Epicoccum* spp., and *Cladosporium* spp.	Not specified	Direct Plating Techniques (PDA) and ITS-amplicon metabarcoding analysis	[52]
Wheat Barley	Lithuania	n = 71	*Fusarium* spp. (*F. avenaceum*, *F. graminearum*), *Alternaria* spp., *Ulocladium* spp., *Penicillium* spp., *Aspergillus* spp., *Bipolaris* spp.	Not specified	Dilution Plate Method (MEA)	[53]
Wheat	Kenya	n = 104	*Epicoccum* spp. (52.8%), *Alternaria* spp. (34%), *Fusarium* spp. (6.4%), *Aspergillus* spp. (<6.4%), *Penicillium* spp. (<6.4)	Not specified	Direct Plate Method (CZA and PDA)	[54]
Wheat	Lithuania	n = 13	*Fusarium* spp. (85%), *Alternaria* spp. (69%), *Penicillium* spp. (54%), *Verticillium* spp. (54%), *Aspergillus* spp., *Mucor* spp., and *Rhizopus* spp.	3.2–5.8	Dilution Plate Method (PDA)	[55]
Wheat	Germany	Not specified	*Fusarium* spp. (*F. culmorum*, *F. graminearum*, and *F. poae*)	Not specified	Not specified	[56]
Barley and Wheat	Denmark	n = 500	*Penicillium* spp., *Alternaria* spp., *Eurotium* spp., *Aspergillus* spp.	Not specified	Direct Plating Techniques	[57]
Wheat	Egypt	n = 20	*Aspergillus* spp., *Alternaria alternata*, *Cladosporium cladospoioides*, *Epicoccum nigrum*, *Penicillium chrysiogenum*, and *Rhizopusnigricans*.	0.08–0.86	Dilution Plate Method (CZA)	[58]
Wheat	Algeria	n = 200	*Alternaria alternata*, *Alternaria infectoria*, *Fusarium acuminatium*	Not specified	Surface Disinfection and Direct Plate Method (PDA)	[59]
Wheat	India	Not specified	*Aspergillus terreus*, *Aspergillus oryzae*, *Aspergillus glaucus*, and *Syncephalastrum racemosum*	Not specified	Dilution Plate Method (SDA)	[60]
Wheat	Poland	n = 22	*Fusarium* spp. (95.5%), *Aspergillus* spp. (81.8%), *Penicillium* spp. (72.3%), *Alternaria* spp. (22.7%), *Mucor* spp. (4.5%)	2.30–5.04	Dilution Plate Method (GKCH)	[61]
Wheat	Slovakia	n = 48	*Fusarium* spp. (70.5%), *Penicillium* spp. (68,2%), *Aspergillus* spp. (61.4%), *Cladosporium* spp. (45.5%), *Alternaria* spp. (34.1%), *Mucor* spp. (27.3%)	2.8–3.8	Dilution Plate Method (GKCH)	[61]
Rye	Poland	n = 23	*Fusarium* spp. (86.9%), *Aspergillus* spp. (73.9%), *Penicillium* spp. (78.3%), *Cladosporium* spp. (30.4%), *Mucor* spp. (17.4%), *Alternaria* spp. (13.0%),	2.5–4.6	Dilution Plate Method (GKCH)	[61]
Rye	Slovakia	n = 4	*Fusarium* spp. (75.0%), *Aspergillus* spp. (25.0%), *Penicillium* spp. (50.0%), *Alternaria* spp. (75.0%)	3.30–3.73	Dilution Plate Method (GKCH)	[61]
Barley	Slovakia	n = 8	*Penicillium* spp. (75.0%), *Alternaria* spp. (66.7%), and *Cladosporium* spp. (58.3%)	3.11–3.69	Dilution Plate Method (GKCH)	[61]
Wheat	Iran	n = 34	*Alternaria* spp. (26.7%), *A. niger* (21.4%), *Fusarium* spp. (17.8%), *A. flavus* (10.7%), *Cladosporium* spp. (6%), *Penicillium* spp. (8.9%), *Rhizopus* spp. (3.5%)	Not specified	Dilution Plate Method (SDA)	[62]
Barley, oats, rye, wheat, millet	Brazil	n = 45	*Aspergillus flavus* (11.9%), *A. amstelodami* (10.4%), *P. polonicum* (10.4%), and *Penicillium citrinum* (9%)	0.43	Direct Plating Techniques (DG 18 and DRBC)	[63]
Maize	Ethiopia	n = 150	*Aspergillus* spp. (75%), *Fusarium* spp.(11%), *Penicillium* spp. (8%), and *Trichoderma* (6%)	Not specified	Dilution Plate Method (PDA)	[64]
Maize	Uganda	n = 256	*A. flavus*, *A. paraciticus*, *and A. tamarii*	0–5	Dilution Plate Method (MRBA)	[65]
Wheat and Barley	Morocco	n = 15	*Aspergillus* spp, *Penicillium* spp., *Fusarium* spp., *Alternaria* spp.	Not specified	Direct Plating Techniques (CZA)	[44]

^1^ Main fungal contaminants reported and frequency data reported in %. 2 Quantitative data reported in variable units, as reported by various research studies.

### 3.2. Main Fungal Contaminants in Flour and Bread

Flour and bread are susceptible to fungal contamination, resulting in their spoilage, inducing economic decline, reducing their shelf-life, and increasing food wastage [10]. In Germany, it was reported that about 35% of all baked products end up being wasted [66]. Consequently, awareness of the fungal load and degree of contamination in flour and bread has greatly increased. Such fungal contamination, is of great concern to producers, manufacturers, and authorities who work tirelessly to protect the health of the consumer. The main stage of fungal contamination occurs during the above-mentioned manufacturing steps, including pre- and post-harvest, transportation, and processing. Contamination of such produce is inevitable since elements, including air, water, soil, and dust, promote permanent and ubiquitous fungal spore presence [67]. The mycoflora of the raw, intermediate ingredients, and final product depend on different factors, such as geographical location, seasonal climate conditions, precipitation level, relative humidity level, product formulation, and processing method [68,69]. It is especially important to characterize and closely monitor the mycological contamination in raw ingredients since these could result in carry-over contamination in freshly made bakery products and, hence, cause fungal spoilage issues [70]. Through the linkage of various stages within the bread processing chain, one can connect important trends to identify common fungal contaminants causing spoilage. It is already known that the mycological inoculum present in agricultural crops, such as cereals and grains, is relatively high [68]. Questions are constantly being raised by the bakery industry if the level of inoculum decreases or increases in derived raw materials, such as flour, and if the latter product is stable during the storage phase. These concerns give rise to further mycological assessments and quality assurance of food products, especially for those within the baking industry [71]. Various types of flour are used to produce different forms of bread, such as white, wholegrain, sourdough, flatbread. When flour is mixed, it can release airborne fungal contaminants, potentially leading to the deposition of these contaminants on surfaces, causing further cross-contamination [68,72,73]. Whole wheat flour and products derived from it are at a higher risk of fungal contamination than any other flour products. It should be noted, however, that flour does not support fungal growth when the water activity (*a_w_*) present is low (*a_w_* < 0.60) [74]. Nonetheless, when storage conditions change, and the moisture levels increase above 12%, such microorganisms, especially xerophilic molds, such as *Fusarium* and *Alternaria*, tend to flourish [71]. Garcia et al., (2019) [68] identified that bread-making raw materials, such as corn flour, had significant fungal contamination of known spoilers, including *Aspergillus* spp. and *Penicillium* spp. The same study also identified that a common fungal spoiler, *Penicillium roqueforti*, was prevalent in all bread types and raw materials used. *P. roqueforti* was also isolated from the air of the same bakery facility in cold processing and storage areas, leading to the conclusion that dispersion of aerosols within baking facilities, including that of flour particles, result in the deposition of fungal contaminants on the surfaces of equipment and fresh baked goods. Santos et al. (2016) remarkably detected fungal species in 100% of all whole flour (wheat and corn) samples tested, notably *Penicillium polonicum* (16.8%), *Aspergillus candidus* (15.2%), *Penicillium commune* (8.8%), *Fusarium* spp. (28.6%), and *Aspergillus flavus* (11.9%) [63]. In this study, the aforementioned species isolated from flour were associated with bread spoilage. The frequent fungal contamination phenomena described above have an impact on the generated bakery products. Bread is a great medium for fungal growth due to its porous structure and adequate supply of oxygen [75]. Conventionally, bread has an *a_w_* of around 0.95 and high moisture content, with a pH of 6, making it extremely vulnerable to fungal contamination [76]. Freire (2011) calculated that approximately 10% of bread produced in Brazil was lost due to fungal spoilage [27]. Losses are suffered not only in finished products but also in early stages, as in the United States, $300 million are lost each year due to wheat fungal spoilage and the production of mycotoxins [37]. Associated genera of known fungal spoilers in bakery products are *Penicillium* spp., *Aspergillus* spp., *Wallemia* spp., *Cladosporium* spp., *Mucor* spp., *Rhizopus* spp., and *Neurospora* spp. [68,73].

Table 2 compiles fungal contaminants from numerous published research papers that reported the occurrence or prevalence of fungal contaminants in flour. Briefly, the most common technique adopted to isolate the fungal contaminants from flour was the direct plating technique. The latter provides a quick screening analysis of what the contaminant is, and, therefore, links the potential mycotoxin that may be present. Hence, the attention is more focused on the quantification of the level of mycotoxin present in the raw material. Fungal contaminants isolated from flour were more variable than what was reported from the grains in Table 1.

### 3.3. Methods of Detecting Fungal Contaminants in Grains/Flour

Methods of identification of fungal contaminants are based on culture-dependent and culture-independent techniques. Culture-dependent techniques rely on a much more classical approach where selective and enrichment media are used to encourage the growth of such contaminants. Potato Dextrose Agar (PDA), Dichloran Glycerol (DG-18) Agar, Malt Extract Agar, Czapek’s Agar (CZA) mediums are most commonly used. Some studies would prefer to perform direct inoculation of the kernel or the flour onto the media, while some studies prefer preparing a homogenate containing a known volume of ringer’s solution or peptone water and amount of flour in grams and then inoculating the homogenate on the media accordingly. Other studies prefer to first disinfect the surface of the kernels using either 70% alcohol or 1.0% sodium hypochlorite (NaOCl) and then inoculating the kernels onto the media followed by incubation at 25 °C for 5 days. Enumeration of colony forming units (CFU) is performed and reported in CFU/g. Purity plates are inoculated in order to perform macroscopic and microscopic assessment studies to attempt to identify the organisms contaminating the grains or flour. Conventionally, wet mounts are prepared for the pure cultures, using a microscopic slide and lactophenol cotton blue (LPCB) as the staining solution. The prepared mounts are then observed under the microscope, where the organisms are identified through distinctive morphological features.

In a more novel and recent approach, culture-independent techniques are being adopted to study the mycobiome of what is contaminating our food and, therefore, uncover a broad spectrum of organisms that either could not be identified through conventional techniques or are unculturable. Target-gene amplicon sequencing is the most exploited high-throughput sequencing application in fungal ecology. As for fungi, the most commonly used target is the internal transcribed spacer (ITS) through the 18S gene part.

Metabarcoding techniques still offer a lot of challenges to researchers since bioinformatics tools are still not considered highly advanced. Minutillo et al. [39] discussed that some operational taxonomic units (OTUs) were still not able to be identified during amplicon target metabarcoding due to library preparation tools being much shorter than commonly used for fungal isolates. Hence, some OTUs were grouped at the genus level so as not to report incorrect data. However, the author admitted that by using metabarcoding datasets, a larger portion of fungal organisms were uncovered, and, therefore, fungal diversity was characterized. In vitro culture techniques require cells that are viable and alive, while metabarcoding techniques may ignore the viability of the fungal cells, and taxonomy data still crop up regardless of whether the cells are alive or dead. Metabarcoding enumeration data rely on the relative abundance of each fungal taxon within the sample being run, and as a result, one has to be careful on how to interpret such quantitative data because sometimes the rRNA markers of different fungal taxa are not PCR-amplified within the same efficiency due to numerous factors, such as primer set, specificity, PCR reagents, and reaction temperatures. Therefore, despite the fact that metabarcoding is a very powerful technique, relative abundance results can mislead the researcher into thinking that the latter are representative of the fungal diversity present within the mycobiome.

## 4. Fungal Toxins in Cereal Grains and Flours—Mycotoxins

### 4.1. Mycotoxins Contaminating Cereals and Their Sources (Fungi)

Till now, mycotoxins of concern to European legislation [88] present in cereals and their products include the four (B1, B2, G1, G2) aflatoxins (AFs), ochratoxin A (OTA), two (B1, B2) fumonisins (FBs), zearalenone (ZEN), deoxynivalenol (DON) also known as vomitoxin, Citrinin (CIT), Ergot alkaloids (EAs), and T-2 and HT-2. The above-mentioned mycotoxins have been considered dangerous, and it is encouraged that simultaneous analysis of them be performed to detect co-occurrence. Table 3 cites the major regulated or registered as carcinogenic from IARC mycotoxins and producing species in cereal grains as reported in the literature. Fumonisins B3, B4, and nivalenol (NIV) are also frequently detected in cereals, although no limits exist to regulate their allowable levels. On the other hand, patulin, detected mainly in fruits, is now present in fruit-based cereal products [89].

As observed in Table 3, the most important mycotoxins in cereals and their products are produced by different species of eight genera of fungi: *Aspergillus*, *Alternaria*, *Byssochlamys*, *Claviceps*, *Fusarium*, *Neotyphodium*, *Paecilomyces*, *and Penicillium*. However, species of the *Fusarium* genus are responsible for the contamination of cereals with DON, FBs, HT-2, T-2, and ZEN, but also with beauvericin (BEA), asenniatins (ENNs), fusaproliferin (FUS), moniliformin (MON), NX-2 toxin, and NIV, whereas those of *Aspergillus* for contamination with AFs and Sterigmatocystin. Alternariol (AOH) is produced by fungi species of the genus *Alternaria*. On the other hand, one mycotoxin can be produced by a variety of fungi. For example, patulin is a secondary metabolite of several species of fungi of the genera *Penicillium*, *Aspergillus*, and *Byssochylamys*, OTA and CIT of the genera *Penicillium*, and *Aspergillus*, FBs of *Fusarium* and *Aspergillus* whereas EAs of *Claviceps* and *Neotyphodium.* On the other hand, toxigenic fungi species can produce more than one type of mycotoxin; therefore, the co-existence of more than one mycotoxin on the same substrate could usually be noticed [90].

Advances in analytical techniques and equipment allowed the determination of many toxic fungal metabolites contaminating cereals and their derived products. Mycotoxins such ENNs, BEA, MON, FUS, alternariol (AOH), sterigmatocystin (STC), and NX-2 toxin, are receiving increased attention due to their high frequency of occurrence and levels of contamination in cereals [55,56,57]. These toxins, although reported in the literature more than one decade ago [91,92,93,94,95], are not routinely determined, and not legislatively regulated, and are still considered “emerging mycotoxins” even though literature reports increasing incidence in the cereals. On the other hand, the small number of investigative studies dealing with the occurrence of these mycotoxins and/or the lack of toxicity data impede risk assessment and the dietary exposure of humans to these mycotoxins [96,97,98].

Some mycotoxins are produced by fungi that colonize the host plant (fungal metabolite) and are released into the cereals, while others are modified mycotoxins, either plant-made metabolites or process-made, through the chemical reaction in the food matrix during food processing. The plant-made metabolites can be divided into two groups, involving the free (extractable) and the bound to other molecules form of modified mycotoxins recognized as “masked mycotoxins” (or conjugated). Most of these modified mycotoxins are considered “emerging mycotoxins”, and awareness about them is increasing.

### 4.2. Alternaria Genus and Its Toxic Metabolites

Although alternariol (AOH) is the most prominent mycotoxin produced by the genus *Alternaria*, it can produce a wide variety of toxic metabolites that are now getting the attention of scientists. They can be classified into five different structural groups. The first is the group of the dibenzopyrone derivatives to which the AOH belongs, alternariol monomethyl ether (AME), and altenuene (ALT). The second group includes the perylene derivative, including altertoxins I, II, and III (ATX-I, ATX-II, and ATX II), alterperylenol (ALTP), and stemphyltoxins (STE), and the third is a tetramic acid derivatives group that comprises tenuazonic acid (TeA) and iso-tenuazonic acid (iso-TeA). The fourth group comprises TA1, TA2, TB1, and TB2 toxins (AAL TA1, TA2, TB1, and TB2) of *A. alternata f*. sp. *lycopersici*, and in the fifth group, arecyclic tetrapeptide toxins tentoxin (TEN), iso-tentoxin (iso-TEN), and dihydrotentoxin (DHT) [99,100,101]. Among them, AOH, AME, ALT, and TeAwere most frequently studied. Tebele et al. [102] reported the presence of AME and TeA in cereals. whereas Gotthardt et al. [103] reported the presence of AOH, AME, TEN, ATX-I, ALTP, and TeA in cereal food for infants and young children. Of them, AOH and AME have been recognized as genotoxic in mammalian cells in vitro [104]. According to EFSA’s opinion on the risks for animal and public health related to the presence of *Alternaria* toxins in feed and food, taking into consideration AOH, AME, ALT, TEN, TeA, altertoxins, STE, and *Alternaria alternata f. splycopersici* toxins, there is need for additional toxicity and occurrence data [105,106]. Besides the aforementioned mycotoxins produced by *Alternaria* spp. in cereals, other toxic metabolites, such as macrosporin and radicinin, are observed [107].

### 4.3. Fusarium Genus and Its Toxic Metabolites

*Fusarium* mycotoxins, frequently detected in cereals and cereal-based products, are predominantly zearalenones, trichothecenes, and fumonisins.

Zearalenone (ZEN) is the main mycotoxin of the group of zearalenones, considered possibly carcinogenic (IARC Group 3), present in cereals and has estrogenic effects [108]. Moreover, its derivatives, α-zearalenol (α-ZEL) and β-zearalenol (β-ZEL), have also been detected in cereals [102].

Trichothecenes present in cereals, on the other hand, are classified in the Type A and Type B groups. Type B trichothecenes group are the most frequently occurring mycotoxins in cereals and include DON and NIV (both considered Group 3 according to IARC) [108] and their acetylated derivatives 3ADON (3-acetyldeoxynivalenol), 15ADON (15-acetyldeoxynivalenol), and 4ANIV (4-acetylnivalenol), respectively. On the other hand, the new type, A trichothecenes group besides T-2 toxin (Group 3, [108]), HT-2 toxin, neosolaniol (NEO), and diacetoxyscirpenol (DAS) toxins, comprises NX-2, NX-3, NX-4, NX-5, and NX-6 that can also be produced in cereals [109,110]. Varga et al. (2015) [109] reported the production of NX-2trichothecene mycotoxin in rice cultures and its deacetylated form NX-3 in wheat. They tested the toxicity of these mycotoxins and noted that NX-3 inhibits protein biosynthesis to the same extent as deoxynivalenol, while NX-2 is far less toxic, similar to 3-ADON. Although detected in low amounts in cereals and their products, under continuous changes in climate and agronomic practices, their presence should no longer be regarded as negligible [111]. Moreover, *F. culmorum* strains are able to simultaneously produce NX-2 with 3-ADON and DON or NIV [112]. New, less-toxic toxins belonging to A trichothecenes group, named NX2-M1, and the related acetylated compounds (NX3-M1 and NX4-M1), representing the degradation products encountered during cereal processing, have also been isolated in processed cereals recently [102]. The group of Type-B trichothecenes that are present in cereals and their products [113,114] comprises fusarenon-X (FUS-X), considered Group 3 by IARC [108]. Moreover, 4,15-diacetoxyscirpenol (DAS), neosolaniol (NEO), and verrucarol (VOL) are other mycotoxins of type-A trichothecene group produced by several *Fusarium* species reported in cereal grains and cereal-based products [115,116].

Fumonisins (FBs), produced mainly by the species *F. verticillioides* and *F. proliferatum*, have been divided into four categories (A, B, C, and D), with B containing, among others, the most toxic compounds. B-group fumonisins comprise fumonisin B1 (FB1), fumonisin B2 (FB2), and fumonisin B3 (FB3). FB1 and FB2 are considered potential human carcinogens (Group 2B) IARC [108] and IARC [117], respectively. Since these toxins can be present contemporarily in cereal commodities, the regulated limits in the EU include the sum of them (FB1 + FB2).

*Fusarins* are another group of mycotoxins produced by *Fusarium fungi*, such as *F. avenaceum*, *F. culmorum*, *F. fujikuroi*, *F. graminearum*, *Fusarium oxysporum*, *Fusarium poae*, *Fusarium sporotrichioides*, and *Fusarium venenatum* [118]. Among the different fusarins (A, B, C, D), fusarin C is the most isolated and identified type in cereals. It is biosynthesized by several *Fusarium* fungi species that contaminate cereals and is classified as possibly carcinogenic Group 2B by IARC [108].

There are also *Fusarium* species that do not produce zearalenones, trichothecenes, fumonisins, or fusarins but produce instead enniatins (ENs), beauvericin (BEA), and moniliformin (MON). These mycotoxins, together with fusaproliferin (FUS), may contaminate cereals [119]. *Fusarium* species *F. acuminatum*, *F. arthrosporioides*, *F. avenaceum*, *F. tricinctum*, *F. torulosum*, *F. kyushuense*, *F. poae*, *F. sporotrichioides*, *F. venenatum*, *F. compactum*, *F. proliferatum*, *F. subglutinans*, *F. verticillioides*, *F. temperatum*, and *F. ramigenum* are reported to produce ENs in cereals [120]. *Fusarium* species that produce beauvericin are reported to be the following: *F. subglutinans*, *F. bulbicola*, *F. denticulatum*, *F. lactis*, *F. phyllophillum*, *F. pseudocircinatum*, and *F. succisae* [121,122]. Several *Fusarium* species, such as *F. avenaceum*, *F. proliferatum*, *F. subglutinans*, *F. oxysporum*, *F. chlamydosporum*, and *F. anthophilum* produce MON, whereas, although named after it, only a few of the strains of *F. moniliforme* can produce it [123]. MON is one of the main *Fusarium* toxins in cereal and is less toxic than the T-2 toxin [124].

ENN and BEA that belong to the group of cyclic hexadepsipeptides are detected in food and unprocessed grains [119,125,126]. In cereals, only seven enniatins (enniatins A, A1, B, B1, B2, B3, and B4) have been detected, although naturally, they exist in a much higher number. Of them, most frequently, only four enniatins (A, A1, B and B1) have been detected [97], with ENB as the most detected enniatin [114,127]. Eniatins A, A1, B, and B1 in cereals are produced mainly by *F. avenaceum*, *F. tricinctum*, and *F. poae* [128]. On the other hand, in cereals is present FUS [119,125], a toxic bicyclic sesterterpene produced by *F. proliferatum*, *F. subglutinans*, *F. antophilum*, *F. begoniae*, *F. bulbicola*, *F. circinatum*, *F. concentricum*, *F. succisae*, *and F. udum* [129,130,131]. According to [96], beauvericin, enniatins, and moniliformin regularly co-occur in cereal grains with other *Fusarium* toxins, such as deoxynivalenol and fumonisins. Other less-known *Fusarium* mycotoxins include metabolites equisetin and butanolide [132].

### 4.4. Aspergillus and Penicillium Genus and Their Toxic Metabolites

Besides the aflatoxins, ochratoxins (OTA), patulin, and sterigmatocystin (STC), the fungi of the *Aspergillus* genus can also produce other toxins in cereals. *Aspergillus* mycotoxins AFs (B1, B2, G1, G2) got great attention due to their potent toxicity (Group 1, [117]). Patulin is classified as a Group 3 carcinogen according to IARC [133]. On the other hand, sterigmatocystins are considered a penultimate precursor of aflatoxins B1 and G1 [134,135] and are defined as a possible human carcinogen (Group 2B) according to the International Agency for Research on Cancer classification [133]. Because of climate change, this mycotoxin is considered a high risk of exposure for consumers [136]. According to EFSA CONTAM Panel [97], there is limited data about STC occurrence in food to assess human dietary exposure. It was noticed that certain strains of *A. niger*, a very important industrial microorganism, produce fumonisin B2, whereas others can produce both ochratoxin A and fumonisins, contaminating foods with both types of carcinogenic mycotoxins [137]. Besides STC, ochratoxin B (OTB) and cyclopiazonic acid (CPA) are also present in cereals [17] but have received much less attention. CPA, ochratoxins, and citrinin (CIT) (Group 3, [133]) have been reported to be produced in cereals by several fungus species of two genera, *Aspergillus* and *Penicillium* [123,138,139]. It was recognized that the negative effects of the simultaneous presence of aflatoxins and CPA were cumulative in most cases [135,139]. Among ochratoxins, ochratoxin A (OTA) (Group 2B according to IARC) [108] occurs more frequently in cereals and is considered ten times more toxic than OTB, while ochratoxin C (OTC) is less than OTB [138]. Gliotoxín is another mycotoxin produced by several species of *Aspergillus* (i.e., *A. fumigatus*) in cereals. It is also associated with the presence of fungi from species of other genera, such as *Trichoderma* and *Penicillium* [140,141,142].

Mohammed et al. [107], in their study conducted on sorghum grains reported, the presence of a high number of less-known toxic metabolites; methoxysterigmatocystin, versicolorin C, averufin, 8-O-methylaverufin, kojic acid, 3-nitropropionic acid, asperflavine, asperfuran, asperloxine A, aspochracin, sydonic acid, viomelleinemodin produced by *Aspergillus* species and mycophenolic acid, mycophenolic acid IV, 1-deoxypebrolide, 7-hydroxypestalotin, barceloneic acid, chanoclavin, cycloaspeptide A, cyclopenin, cyclopenol, dechlorogriseofulvin, dehydrogriseofulvin, F01 1358-A, flavoglaucin, griseofulvin, NP1793, O-methylviridicatin, penicillic acid, quinolactacin A, quinolactacin B, PF 1163, rugulovasine A from *Penicillium* species.

### 4.5. Other Fungi Genera and Their Toxic Metabolites

Fungi of the fungal genus *Claviceps*, which causes ergot disease in plants, are recognized to produce toxic ergot alkaloids in cereal crops [143]. EAs are produced by the fungi *C. purpurea*, *C. fusiformis*, and *C. africana* of the genus *Claviceps*. Based on the data collected, EFSA CONTAM Panel [143] suggested monitoring some of the *C. purpurea* EAs. In addition to ergometrine, ergotamine, ergosine, ergocristine, ergocryptine (mixture of α- and β- isomers), and ergocornine, the biologically inactive corresponding -inine epimers were suggested to be monitored because, at different processing conditions, interconversion could occur.

In addition to all the aforementioned mycotoxins, the presence of other, less common fungus genera metabolites was observed in cereals, such as Abscisic acid, Cytochalasin B, Destruxin A, Monocerin, Preussin, Terphenyllin, Terrein, and Trichodermamide C [107].

### 4.6. Conjugated Masked Mycotoxins in Cereals

Plants could decrease the toxicity of certain mycotoxins by utilizing their enzymatic and/or hormone potential to bind them with specific moieties, transforming them biologically. This modification is realized in plants that have developed a defensive mechanism to protect themselves from the deleterious nature of mycotoxins [144,145]. Plants can metabolize mycotoxins utilizing their metabolism following three phases. Phase I comprises the enzymatic transformation of mycotoxins through oxidation, reduction, or hydrolysis; during phase II, there are observed processes such as sulfatation, glucosidation, and glucuronidation [146]; and during phase III (detoxification), the compounds conjugated to glucose or glutathione are confined/attached to the plant cells [147].

Following a series of processes, the mycotoxins’ structure is changed and stabilized by conjugation with glucoside, acetyl, sulfate, and/or glutathione or other macromolecular substances [148]. These modified forms can be both covalently or not covalently bound and are not only restricted in the kernels of cereals but are proven to occur with mild temperature exposure as well as in thermally treated cereal products [149,150]. Mycotoxins after structure transformation are referred to as ‘’modified”, “masked”, or “conjugated” mycotoxins. The transformed mycotoxins are permanently stored in the plant tissue rather than excreted.

The literature reports many mycotoxins such as deoxynivalenol, zearalenone, fumonisins, nivalenol, fusarenon-X, T-2 toxin, HT-2 toxin, ochratoxin A, and patulin to be metabolized or bind by the plants [147,148]. Besides DON and its biologically transformed form, the deoxynivalenol-3-glucoside (DON-3G) [126,147] has been detected. Moreover, α- and β -zearalenone-14-β-D-glucopyranoside (ZEN-14-Glc) is the plant metabolite of zearalenone (ZEA). Another risk in cereals can arise from cis-ZEN, the isomerized form of ZEN, which can be produced as a result of exposure to daylight and can be found as a natural food contaminant [64] together with its cis-form retaining significant estrogenic activity [151]. Streit et al., have detected the presence of zearalenone-4-sulfate in their samples [126]. Beccaccioli et al., suggested that fumonisins produced by *F. verticillioides* alter maize lipid metabolism in order to adapt fungal growth to a relatively harmless destructive form and protect themselves [152]. Fumonisins undergo modification in cereal plants conjugating with fatty acid esters forming fatty acid esters of FBs, thus affecting their analytical detection [149]. These mycotoxins that are not screened routinely in foods are not regulated by legislation. EU Commission [153] recommends, as appropriate, analyzing T-2 and HT2 toxins and their masked mycotoxins, particularly the mono- and di-glycosylated conjugates of T-2 and HT-2 toxins.

The modified mycotoxins have raised the concern of scientists because, inside the human metabolism, they are hydrolyzed to their initial much higher toxic form [154]. Although present in food, they are not detected during routine determinations due to their physicochemical behavior that depends on their different chemical structure.

*Fusarium* species are strongly related to the production of mycotoxins and the contamination of cereals in the field while at the post-harvest stage depending on the storage conditions, species of *Aspergillus* and *Penicillium* are predominant [19,155]. The conditions that favor mycotoxin production include moisture (expressed as either relative humidity (RH) or water activity (a_w_)), temperature, pH, fungal species, substrate, drought stress, insect damage, and mechanical stress of the plants [156,157]. Therefore, a holistic approach should be implemented involving every stakeholder in the food chain to minimize mycotoxin contamination [158].

Due to the modifications in plants and/or during processing, mycotoxin detection is strongly affected by several factors in the experimental setup, resulting in an altered final compliance assessment. Besides the already legislated mycotoxins, the new mycotoxins, and the masked ones, should not be ignored since combined toxicity may be higher than predicted from individual effects. Thus, the cumulative risk assessment must consider each mycotoxin, its derivatives, and its modified forms present in the same sample [159,160].

Of “new emerging toxins” and “masked mycotoxins”, only a few have been identified as toxicologically relevant for public food safety, however, currently, there are no regulations on most of the toxins contaminating cereals in Europe or other regions of the world. Acute exposure to some of these mycotoxins may not indicate concern for human health, but chronic exposure can represent a concern that needs to be investigated. This fact, together with the high consumption of cereals and their products, makes the detection and study of these mycotoxins a primary necessity for food safety.

**Table 3:** Major mycotoxins and fungal species associated with their production in cereal grains.

Mycotoxins	Acronym	Fungal Species	Source
Aflatoxin B1, B2, G1, G2	AFs	*A. flavus*, *A. nomius*, *A. parasiticus*	[19,161,162]
Citrinin	CIT	*P. expansum*, *A. ochraceus*, *P. verrucosum*	[163]
Deoxynivalenol	DON	*F. acuminatum*, *F. culmorum*, *F. graminearum*	[19,110,161,162,164,165]
Ergot alkaloids (EA s)	EAs	*C. purpurea*	[166]
Fumonisin B1, B2, B3, B4	FBs	*F. proliferatum*, *F. verticillioides*, *A. niger*	[137,162,164,165]
HT-2	HT-2	*F. langsethiae*, *F. poae*, *F. sambucinum*, *F. sporotrichioides*	[164,165]
Nivalenol	NIV	*F. cerealis*, *F. crokwellense*, *F. culmorum*, *F. graminearum*, *F. poae*	[110,164,165]
Ochratoxin A	OTA	*A. carbonarius*, *A. ochraceus*, *P. cyclopium*, *P. nordicum*, *P. verrucosum*, *P. viridicatum*,	[19,161,162]
Sterigmatocystin	STC	*A. flavus*, *A. parasiticus*, *A. nidulans*, *A. versicolor*	[167]
T-2	T-2	*F. acuminatum*, *F. equiset*, *F. langsethiae*, *F. poae*, *F. sambucinum*, *F. sporotrichioides*	[161,164,165]
Zearalenone	ZEN	*F. crokwellense*, *F. cerealis*, *F. culmorum*, *F. equiseti*, *F. graminearum*,	[161,162,164,165]

A.: *Aspergillus*, C.: *Claviceps*, F.: *Fusarium*, P.: *Penicillium*.

## 5. Occurrence of Mycotoxins in Wheat Flour

Table 4 provides information on the type of product (wheat flour or wholemeal flour, emphasizing organic cultivation), the country of origin, the number of contaminating mycotoxins, and the analytical methods used for mycotoxin determination in wheat flour. Out of the 69 studies, 64 focused on plain flour, while ten also investigated wholemeal flour. The studies cited were carried out in countries from four continents: Africa (Egypt, Ethiopia, Nigeria), Asia (China, Iran, Japan, Lebanon, Palestine, Pakistan, South Korea, Turkey), Europe (Bosnia and Herzegovina, Croatia, Czech Republic, Hungary, Italy, Poland, Portugal, Romania, Switzerland, The Netherlands), and South America (Argentina and Brazil). The majority of the studies (54%) were from Asia, especially China, contributing to 20% of the total. The EU accounted for another 20%, while 19% were from South American nations.

Several types of mycotoxins have been included in these studies, receiving varying degrees of attention from scientists. DON (36 studies) was the most extensively studied, with 36 research articles focusing on its analysis, while Aflatoxins and Zearalenone followed with 20 studies and Ochratoxins, particularly Ochratoxin A, was the subject of 19 studies. In contrast, Fumonisins, T-2, and HT-2 received relatively little attention. Research teams showed interest in evaluating the presence of novel mycotoxins such as *Alternaria* toxins (AME, AOH, TeA, and TEN), mycotoxins derived from *Fusarium* (BEA, DAS, ENNs, FUS-X, NEO, NIV) and *Aspergillus* genera (CTV, STC). It is worth noting that the determination of mycotoxins was carried out using various methods each with its own level of accuracy and precision. These methods are also discussed in detail in the following section, providing a comprehensive understanding of the analytical approaches employed in the analysis of mycotoxins in a wheat flour substrate.

As previously noted, the trichothecene mycotoxin DON is reported to be the most frequently occurring mycotoxin. Therefore, it attracted significant attention of the research teams that analyzed its main form, its acetylated derivatives 3-acetyl-deoxynivalenol (3AcDON), and 15-acetyl-deoxynivalenol (15AcDON), as well as its masked form, deoxynivalenol-3-glucoside (D3G). The highest concentrations of DON were reported in wheat flour samples from Brazil, with reported levels ranging from 2711 to 3046 μg/kg [168], from 1666 to 5822 [169], and from 73.50 to 2794 [170]. These concentrations significantly exceed the maximum levels established by the European Commission as well as that of the Brazilian regulation, which are 750 and 1000 μg/kg, respectively [171,172]. In China, a systematic study conducted involving 10,192 samples from 30 provinces reported a rather high incidence of DON (77.5%), although at low concentration levels [173]. Lower DON contamination was reported in samples from the EU as well as in Asian countries, particularly Pakistan [174] and China [56]. The lowest level of contamination with DON was observed in samples from African regions [175]. In the work conducted by Gab-Allah et al., 2021, it was observed that the level of contamination with D3G in wheat flour of organic origin was found to be higher compared to conventional wheat flour [113]. This trend of higher contamination in samples of organic cultivation is also noticeable for other mycotoxins. However, the exact reasons behind the increased mycotoxin contamination in organic samples remain unclear. It is uncertain whether this higher contamination is solely attributed to the non-use of fungicides or if other factors, such as climate conditions, crop location and rotation, and tillage practices, may also play a significant role in this phenomenon [176]. Further research is required to gain a deeper understanding of the factors contributing to mycotoxin contamination in organic versus conventional agricultural practices.

In most countries across the globe, for aflatoxins one finds maximum levels regarding aflatoxin B1 (AFB1) set at 2 μg/kg while the total sum of aflatoxins (B1, B2, G1, and G2) [171,177,178,179,180,181,182] vary from 4 μg/kg in EU up to 20 μg/kg in Iran. However, when it comes to baby and infant foods, the levels are much lower, typically not exceeding 0.1 μg/kg AFB1. A high incidence of AFB1 and AFB2, higher than 70%, was reported in samples from Iran [183,184], while 60 samples from Turkey did not report the presence of any aflatoxins [185,186]. In most studies, AFB1 contamination levels were close to 5 μg/kg, but in the study of Shahbazi and Shavisi, AFB1 for the positive samples ranged from 0.2 to 21.9 μg/kg [183].

Among all the studies that investigated the presence of Zearalenone (ZEA) and its derivative structures α- and β-ZAL (Zearalanol), α- and β-ZOL (Zearalenol) and zearalenone (ZAN) met the established maximum limits set globally. These for EC and Japan are 75 μg/kg [171,187], while for Brazil and China, 100 and 60 μg/kg, respectively [172,177]. It is important to emphasize that these variations regarding the maximum limits can create obstacles that could hinder the trade between regions that follow different regulations.

The maximum limits of HT-2 and T-2 have not yet been established at a regulatory level within the EU’s newly established 2023/915 regulation and its predecessor 1881/2006 [88]. Similarly, these limits are not defined in China, Brazil, and Argentina [172,177,188]. However, the existing recommendation 2013/165/EU allows these two mycotoxins expressed as a sum to be up to 50 μg/kg in wheat flour [153]. Across the samples presented in the above studies, the contamination levels of these two trichothecenes were found to be low in EU samples, with concentrations of 3.8 μg/kg.

As shown in Table 3, several fungal genera, e.g., *Fusarium*, are capable of producing multiple mycotoxins [165,166,167,179,180,181]. Palumbo et al., in their work, reported that across 206 studies, co-occurrence of at least two mycotoxins was identified in 55% of the samples examined [90]. However, this percentage could be substantially higher because, generally, a targeted analysis is usually performed aiming mainly at mycotoxins regulated in the legislation. Consequently, the detection of a single mycotoxin could be an indicator of the presence of multiple ones [46]. Some of the studies reported here showed no co-occurrence [189,190,191,192], while others noticed the presence of many mycotoxins in the same matrix [193,194]. Additionally, mycotoxins tend to be mostly present on the outer fractions of the grain kernels, mainly bran [195,196]. This phenomenon was not observed throughout the entire range of wholegrain flour we report herewith, as plain flour samples exhibited notably higher levels of contamination, a fact, that can be misleading and send an inaccurate signal.

Among novel mycotoxins, NIV was the most extensively studied mycotoxin followed by ENNs, DAS, and *Alternaria* mycotoxins. Other mycotoxins that attracted attention were those originating from *Fusarium* genus particularly the four enniatins (ENNs): enniatin A (ENN A), A1 (ENN A1), B (ENN B), and B1 (ENN B1), beauvericin (BEA), and DAS. In a study involving 181 samples obtained from various Chinese provinces, over 91% of the samples tested positively for TeA, TEN, and AME [197]. NIV was prevalent in 57 and 41 samples studied by the teams of Liu and Zhou, respectively [193,198]. Despite the smaller toxicity of ENNs and BEA compared to other *Fusarium* mycotoxins, they are still of interest due to their presence in high concentrations [199]. Particularly in the samples examined by Zhou et al., ENNA1, ENNB, and ENNB1 showed concentration levels higher than 400 μg/kg [100]. Finally, among the studies under consideration in the last decade, 13 of them investigated samples from six Mediterranean countries, namely, Bosnia and Herzegovina, Croatia, Egypt, Italy, Lebanon, and Turkey. These studies primarily focused on quantifying aflatoxins, followed by ochratoxins and zearalenone. Notably, the highest prevalence of mycotoxins was identified in samples of both conventional and organic origin from Croatia, as reported by Vrček et al., 2014, particularly in relation to Zearalenone (ZEA) and Ochratoxin A (OTA).

**Table 4:** Occurrence of mycotoxins between 2013 and 2023 in wheat flours and their method of detection.

Product	Country	Total Samples (*n*) Positive Samples/Positive Samples (%)	Range of Toxins (Unless Stated Differently in μg/kg)	Estimated Average Exposure (ng/kg bw/day)/Estimated Weekly Intake (EWI)/Estimated Daily Intake (EDI) (ng/kg bw/Day)/Probable Daily Intake (PDI) (μg/kg bw/Day)/Estimated Average Exposure (EAE)/Upper Bound-Lower Bound (UB-LB)	Clean-Up	Analytical Method	Reference
Flour	Portugal; The Netherlands	(*n =* 19) ZEA: 9/47	ZEA: 7.4–15.3		Immunoaffinity column	HPLC-FLD	[200]
Flour	Pakistan	(*n =* 18) AFB1: 12/67 Total AFs: 12/67 OTA: 9/50 ZEA: 11/61	AFB1: LOD-6.65 Total AFs: LOD-6.90 OTA: LOD-5.90 ZEA: LOD-53.70	EAE AFB1: 6.42 OTA: 2.14 ZEA: 73.7	Immunoaffinity column	HPLC-FLD	[201]
Flour	Brazil	(*n =* 58) DON: 53/91	DON: 200–1310		Immunoaffinity column	HPLC	[202]
Flour	Brazil	(*n =* 235) FB1: - FB2: - FB3: - HFB1: - DON: 227/97 D3G: 1/0.4 15AcDON: - ZEA: 6/3 α-ZOL: - OTA: - CTV: - AFB1: - AFB2: - T-2: 1/0.4 Total FUMs: - Total DON: 227/97 Total ZEA: 6/3	FB1: - FB2: - FB3: - HFB1: - DON: 53–3186 D3G: 183.6 15AcDON: - ZEA: 17.8–79.2 α-ZOL: - OTA: - CTV: - AFB1: - AFB2: - T-2: 1508 Total FUMs: 4.0–25 Total DON: <LOQ-3186 Total ZEA: <LOQ-79.2		Filtration	HPLC-MS/MS; UPLC-MS/MS	[189]
Flour	Italy	(*n =* 40) AFB1: -/-	AFB1: -		No clean-up	HPLC-FLD	[203]
Flour	Argentina	(*n =* 5) DON: 5/100	AFB1: 0.16–0.38		Immunoaffinity column	HPLC	[204]
Flour	Italy	(*n =* 1) DON: 1/100	Mean DON: 186		Immunoaffinity column	HPLC-MS/MS	[205]
Flour	Argentina	(*n =* 76) FB1: 52/68 FB2: 40/53 FB1 + FB2: 52/68	Mean/Max FB1: 0.28/2.10 FB2: 0.92/17.52 FB1 + FB2: 1.19/18.94		SPE	HPLC-MS/MS	[206]
Flour	Argentina	(*n =* 34) DON: 31/91	Mean/Maximum DON: 243/> 1000		Immunoaffinity column	GC	[207]
Flour	Iran	(*n =* 96) DON: 80/83	DON: 23–1270		No clean-up	ELISA	[208]
Flour	Brazil	(*n =* 24) DON: 17/71	DON: 1666–5822		QuEChERS	HPLC	[169]
Flour	Turkey	(*n =* 12) AFB1: 3/25 AFB2: 1/8 AFG1: 4/33 OTA: 11/92	AFB1: 0.03–0.72 AFB2: - AFG1: 0.03–6.60 OTA: 0.80–3.02		Immunoaffinity column	HPLC-FLD	[209]
Flour	Brazil	(*n =* 200) DON: 200/100 T-2: 27/13.5 ZEA: 102/51	DON: 53–2905 T-2: 500–1506 ZEA: <LOQ		QuEChERS	UPLC-MS/MS	[210]
Flour	Lebanon	(*n =* 50) OTA: 4/8 OTB: - T-2: - HT-2: -	OTA: 0.6–3.4 OTB: - T-2: - HT-2: -		No clean-up	HPLC-MS/MS	[211]
Flour	South Korea	(*n =* 12) DON: 11/92 D3G: 11/92 NIV: 8/67 3AcDON: 3/25 15AcDON: -/- Fusarenon-X: 2/17	DON: 3.81–153.54 D3G: 0.44–17.60 NIV: 0.45–126.23 3AcDON: 2.30–10.32 15AcDON: - Fusarenon-X: 0.8–3.22		Immunoaffinity column; Filtration	HPLC-MS/MS	[113]
Flour	Egypt	(*n =* 50) DON: 28/56 NIV: 17/34 D3G: 12/24	DON: <LOQ-389 NIV: <LOQ-179 D3G: <LOQ-120	PDI DON: 0.53 NIV: 0.23 D3G: 0.21 DON + D3G: 0.74	Immunoaffinity column; Filtration	UPLC	[175]
Flour	Ethiopia	(*n =* 30) OTA: 15/50	Median OTA: 7.20		SPE	HPLC-FLD	[212]
Flour	Turkey	(*n =* 50) DON: 3/6 ZEA: 2/4	DON: 92–151 ZEA: 51.6–54.6	LB-UB DON: 7.0–27.4 ZEA: 2.12–3.20	Immunoaffinity column	HPLC-FLD	[213]
Flour	Egypt	(*n =* 12) AFB1: 7/58	Mean/StDev AFB1: 0.56/0.20		Filtration	HPLC-FLD	[214]
Flour	Poland	(*n =* 113) OTA: 13/12	OTA: 0.7–5.8	EDI OTA: 900,000	Immunoaffinity column	HPLC-FLD	[215]
Flour	China	(*n =* 67) ENNA: 35/52 ENNA1: -/- ENNB: 38/57 ENNB1: 33/49 BEA: 52/78	ENNA: 0.02–0.19 ENNA1: - ENNB: 0.02–0.59 ENNB1: 0.14–1.31 BEA: 0.12–11.1		SPE	UPLC-MS/MS	[216]
Flour	Iran	(*n =* 54) AFB1: 28/52 AFB2: 21/39 AFG1: 9/17 AFG2: 7/13 Total AFs: 28/52	AFB1: 0.34–5.25 AFB2: 0.14–0.91 AFG1: 0.20–0.42 AFG2: 0.10–0.35 Total AFs: 0.34–6.40		Immunoaffinity column	HPLC-FLD	[217]
Flour	China	(*n =* 39) ZEA: 17/44	ZEA: 0.0068–0.0213		No clean-up	LFR	[218]
Flour	Pakistan	(*n =* 76) DON: 36/47	DON: LOD-1890		No clean-up	HPLC	[174]
Flour	Pakistan	(*n =* 22) FB1: 20/91	FB1: LOD-1390		Immunoaffinity column	HPLC-FLD	[219]
Flour	Iran	(*n =* 180) AFB1: 144/80	AFB1: 0.046–0.073		Immunoaffinity column	HPLC-FLD	[184]
Flour	China and imported (Australia, Japan, Russia, USA)	(*n =* 75) DON: 64/85	12.5–1285.4		Immunoaffinity column	HPLC-MS/MS	[220]
Flour	Argentina	(*n =* 54) 15AcDON: - 3AcDON: AFB1: - AFB2: - AFG1: - AFG2: - AME: - AOH: 9/17 DON: 49/91 ENNA: - ENNA1: - ENNB: 2/4 ENNB1: 2/4 FB1: 5/9 FB2: - HT-2: - OTA: - T-2: - TeA: 2/4 ZEA: -	Mean/Max 15AcDON: - 3AcDON: AFB1: - AFB2: - AFG1: - AFG2: - AME: - AOH: 3.3/45.2 DON: 78.8/622.4 ENNA: - ENNA1: - ENNB: 0.3/3.5 ENNB1: 2.1/47.2 FB1: 5 FB2: - HT-2: - OTA: - T-2: - TeA: 0.8/8.3 ZEA: -		Filtration	UPLC-MS/MS	[166]
Flour	Turkey	(*n =* 60) AFB1: -/- AFB2: -/- AFG1: -/- AFG2: -/- Total AFs: -/-	AFB1: - AFB2: - AFG1: - AFG2: - Total AFs: -		Immunoaffinity column	HPLC-FLD; Post-column derivatization	[186]
Flour	Turkey	(*n =* 60) AFB1: -/- AFB2: -/- AFG1: -/- AFG2: -/- Total AFs: -/- OTA: 16/27	AFB1: - AFB2: - AFG1: - AFG2: - Total AFs: - OTA: 0.105–0.918		Immunoaffinity column	HPLC-FLD; Post-column derivatization	[185]
Flour	Brazil	(*n =* 39) AFB1: - AFB2: - AFG1: - AFG2: - DAS: - DON: 39/100 FB1: - FB2: - HT-2: - OTA: - ZEA: 1/2.6	Mean/StDev AFB1: - AFB2: - AFG1: - AFG2: - DAS: - DON: 1049/917.9 FB1: - FB2: - HT-2: - OTA: - ZEA: 1		QuEChERS	UPLC-MS/MS	[191]
Flour	China	(*n =* not specified) DON: T-2 and HT-2:	DON: 260 T-2 and HT-2: -		Filtration	HPLC-MS/MS	[221]
Flour	China	(*n =* 369) FB1: 23/6 FB2: -/- FB3: -/-	FB1: 0.3–34.6 FB2: - FB3: -		Filtration	UPLC-MS/MS	[222]
Flour	China	(*n =* 359) DON: 349/97 3AcDON: 40/11 15AcDON: 51/14 D3G: 120/33 NIV: 145/40 DOM: -/- FUS-X: -/- ZEA: -/-	DON: 1.3–825.9 3AcDON: 0.6–3.6 15AcDON: 2.0–11.1 D3G: 0.2–15.7 NIV: 0.4–23.9 DOM: - FUS-X: - ZEA: -	EDI DON: 0.385 3AcDON: 0.004 15AcDON: 0.016 D3G: 0.015 NIV: 0.016	Immunoaffinity column	UPLC-MS/MS	[223]
Flour	China	(*n =* 95) ENNA: 19/20 ENNA1: 6/6 ENNB: 41/43 ENNB1: 46/48 BEA: 67/71	ENNA: 0.11–3.30 ENNA1: 0.30–1.78 ENNB: 0.23–18.20 ENNB1: 0.16–33.90 BEA: 0.15–47.10		SPE	UPLC-MS/MS	[224]
Flour	China	(*n =* 348) AFB1: 77/22 AFB2: 1/0 DON: 318/91 15AcDON: 119/34 3AcDON: 11/3 D3G: 19/5 ZEN: 46/13 NIV: 57/16	AFB1: <0.10–7.3 AFB2: <0.10–1.15 DON: <0.10–1129 15AcDON: <0.10–6.0 3AcDON: <0.10–2.6 D3G: <0.25–3.9 ZEN: <0.25–98.8 NIV: <0.20–19.1	PDI AFB1: 0.030 AFB2: 0.004 DON: 0.860 15-Ac-DON: 0.010 3-Ac-DON: 0.010 D3G: 0.010 ZEN: 0.010 NIV: 0.010	Multifunctional cartridges	HPLC-MS/MS	[193]
Flour	China	(*n =* 672) 15AcDON: 121/18 3AcDON: 11/2 DON: 323/48	15AcDON: 0.62–6.0 3AcDON: - DON: 2.4–1130	PDI 15AcDON: 156 3AcDON: 0.87 DON: 0.86	Immunoaffinity column; Multifunctional cartridges	HPLC-MS/MS	[225]
Flour	Brazil	(*n =* 415) DON: 196/47	Mean/Maximum DON: 693/11,400		No clean-up	HPLC-MS/MS	[226]
Flour	Iran	(*n =* not specified) AFB1: -/-	Mean AFB1: 5.35		Immunoaffinity column	HPLC-FLD; Post-column derivatization	[227]
Flour	South Korea	(*n =* 34) T-2: -/- HT-2: 16/47	T-2: - HT-2: 7.1–118.8	EDI HT-2 and T-2: 2.45	Immunoaffinity column	HPLC-FLD	[228]
Flour	Nigeria	not specified	AFB1: 0.73		No clean-up	ELISA	[86]
Flour	Czech Republic	(*n =* 36) DON: 8/22 T-2 and HT-2: -/- ZEA: 2/6	Mean/Max DON: 17.1/76.4 T-2 and HT-2: -/- ZEA: 0.31/0.76	LB-UB DON: 0.41–5.65 T-2 and HT-2: - ZEA: 0.00–0.13	QuEChERS	HPLC-MS/MS	[229]
Flour	Croatia	(*n =* 9) AFB1: 1/11 OTA: 2/22 DON: 6/67 ZEA: 3/33 FUM: 4/44	AFB1: - DON: 27.1–126 OTA: 1.84–2.05 FUM: 35.2–62.3 ZEA: 5.70–10.1		Filtration	ELISA	[176]
Flour	Croatia; Bosnia and Herzegovina	(*n =* 9) T-2 and HT-2: -			No clean-up	ELISA	[230]
Flour	Argentina	(*n =* 100) AOH-3-S: -/- AOH: 13/13 AME: 40/40 TeA: 31/31 ALP: 36/36 ATX-I: 20/20 AME-3-S: 1/1 TEN: 18/18	AOH-3-S: - AOH: <LOQ-13 AME: 0.1–3.3 TeA: <LOQ-330 ALP: <LOQ-47.9 ATX-I: <LOQ-5.6 AME-3-S: - TEN: <LOQ-5.5		Filtration	HPLC-MS/MS	[231]
Flour	Iran	(*n =* not specified) DON: D3G:	Mean/StDev DON: 227/9 D3G: 10/1	EDI D3G: 48 DON: 703	Immunoaffinity column	HPLC	[232]
Flour	Palestine	(*n* = 6 OTA; *n* = 12 Fumonisins; *n* = 17 Total AFs) OTA: -/- Fumonisins: -/- Total AFs: 7/41	Mean Total AFs: 2.8		No clean-up	LFR	[233]
Flour	Iran	(*n =* 60) AFB1: 60/100 AFB2: 44/73 AFG1: 8/13 AFG2: 5/8 OTA: 46/77	AFB1: 0.8–21.9 AFB2: 0.7–5.6 AFG1: 0.4–2.0 AFG2: 0.3–0.6 OTA: 7.8–22.3		Immunoaffinity column	HPLC-FLD	[183]
Flour	Brazil	(*n =* 172) DON: 134/78	73.50–2794.63		Immunoaffinity column	UPLC	[170]
Flour	Kosovo	(*n =* 81) OTA: 4/5	0.26–2.75		No clean-up	ELISA	[234]
Flour	Romania	(*n =* 41) BEA: - ENNA: - ENNA1: - ENNB: 12/29 ENNB1: 2/5	Mean/Max BEA: - ENNA: - ENNA1: - ENNB: 1.8/38.2 ENNB1: 0.5/16.6	EDI BEA: 0.5–3 ENA: 0–31.7 ENA1: 0–10.7 ENB: 25.8–27.8 ENB1: 5.4–10.3 Sum of ENs: 31.2–80.5	Filtration	HPLC-MS/MS	[235]
Flour	Romania	(*n =* 41) 3AcDON: - 15AcDON: 4/10 DON: 17/41 NIV: - FUS-X: - NEO: - DAS: - HT-2: - T-2: - ZEA: -	Mean/Max 3AcDON: - 15AcDON: 2.4/32.6 DON: 147/1947 NIV: - FUS-X: - NEO: - DAS: - HT-2: - T-2: - ZEA: -	EDI DON: 665–666 3AcDON: 0–6.5 15AcDON: 4.4–18	Filtration	GC-MS/MS	[236]
Flour	Hungary	(*n =* 38) 3AcDON: 1/3 15AcDON: 1/3 D3G: - DON: 35/92 DOM: - HT-2: 1/3 T-2: 1/3 AFB1: - FB1: - OTA: - ZEA: - DAS: -	3AcDON: - 15AcDON: - D3G: - DON: 57–318 DOM: - HT-2: - T-2: - AFB1: - FB1: - HT-2: - OTA: - ZEA: - DAS: -		Filtration	HPLC-MS/MS	[192]
Flour	China	(*n =* 10,192) DON: 7899/78	Mean DON: 251		Not specified	HPLC-MS	[173]
Flour	UK and Germany	(*n =* 214) DON: 9/4 OTA: 20/9 T-2/HT-2: 10/5 ZEA: 17/8	Mean/St. Error DON: 48/7 OTA: 2.9/0.1 T-2/HT-2: 1.7/0.4 ZEA: 3.9/0.2		Filtration	LFR; ELISA	[237]
Flour	Japan	(*n =* 50) DON: 44/88 ZEA: 9/18	Mean/Max DON: 71.8/789 ZEA: 1.2/3.3		Immunoaffinity column	HPLC-MS/MS	[238]
Flour	Japan	(*n =* 163) DON: 159/98 ENNA: 2/1 ENNA1: 29/18 ENNB: 137/84 ENNB1: 77/47 BEA: 1/1 NIV: 42/26	Mean/Max DON: 68.1/386 ENNA: 0.1/4 ENNA1: 1.5/27.4 ENNB: 43/633 ENNB1: 7.2/96.4 BEA: 0.3/3.4 NIV: 2.1/43		Immunoaffinity column	HPLC-MS/MS	[239]
Flour	Japan	(*n =* 101) diANIV: -/- diHDAS: -/- HDAS: -/- DAS: -/- NEO: -/- 4β,8α,15-triacetoxy-3α,7α-dihydroxy-12,13-epoxytrichothec-9-ene: -/- T-2: 9/9 HT-2: 26/26	Mean/Max diANIV: -/- diHDAS: -/- HDAS: -/- DAS: -/- NEO: -/- 4β,8α,15-triacetoxy-3α,7α-dihydroxy-12,13-epoxytrichothec-9-ene: -/- T-2: 0.04/1 HT-2: 0.4/4		No clean-up	HPLC-MS/MS	[240]
Flour	Japan	(*n =* 133) STC: 42/32	Mean/Max 0.02/2.4		Immunoaffinity column	HPLC-MS/MS	[241]
Flour	Pakistan	(*n =* 30) AFB1: 10/33	AFB1: 1.83–2.01		No clean-up	TLC	[242]
Flour	China	(*n =* 85) 15AcDON: 7/8 3AcDON: 13/15 DON: 66/77 ZAN: - ZEA: 6/7 α-ZAL: - α-ZOL: - β-ZAL: - β-ZOL: -	15AcDON: 3.24 3AcDON: 7.14 DON: 308.9 ZEA: 1.22		Immunoaffinity column	HPLC-FLD	[243]
Flour	China	(*n =* 181) TeA:180/99 AOH: 11/6 AME: 165/91 TEN: 176/97	TeA: 1.76–520 AOH: 16.0–98.7 AME: 0.320–61.8 TEN: 2.72–129	EAE TeA: 175 AOH: 3.56–24.0 AME: 6.09 TEN: 54.5	SPE	UPLC-MS/MS	[197]
Flour	China	(*n =* 80) TEN: 58/73 DON: 77/96 3AcDON: 8/10 15AcDON: -/- ENNA:53/60 ENNA1: 58/73 ENNB: 70/88 ENNB1: 66/83 OTA: 18/23 OTB: -/- AME: 62/78 BEA: 64/80 DAS: 13/16 NIV: 12/15 TEN: 43/54 AFB1: 12/15 AFB2: 10/13 AFG1: 4/5 AFG2: 3/4 ChA: -/- Penicillic Acid: -/-	TEN: 0.1–30.2 DON: 14.3–2123.6 3AcDON: 12.1–85.6 15AcDON: - ENNA: 0.7–259.3 ENNA1: 5.3–406.4 ENNB: 4.5–822 ENNB1: 2.4–587.7 OTA: 0.12–5.6 OTB: - AME: 0.2–12.6 BEA: 0.3–67.7 DAS: 0.5–9.6 NIV: 14.6–64.7 TEN: 0.16–24.0 AFB1: 0.1–5.2 AFB2: 0.1–1.2 AFG1: 0.1–0.5 AFG2: 0.1–0.2 ChA: - Penicillic Acid: -		SPE	UFLC-MS/MS	[194]
Flour	China	(*n =* 299) DON: 244/75 15AcDON: 113/38 3AcDON: 12/4 D3G: 96/32 FUS-X: 35/12 NIV: 41/14 AOH: 55/18 TEN: 165/55 TeA: 219/73 ZEA: 120/40 FB1: 2/1 OTA: 8/3 NEO: 7/2	DON: 0.8–371.4 15AcDON: 0.8–140.6 3AcDON: 1.6–10.8 D3G: 1.6–96.3 FUS-X: 3.6–191.7 NIV: 3.8–96.7 AOH: 0.2–140.8 TEN: 0.04–14.8 TeA: 0.8–161.6 ZEA: 0.2–5.7 FB1: 31.2–1260.4 OTA: 0.2–1.0 NEO: 0.1–2.6		Filtration	UPLC-MS/MS	[198]
Flour	Croatia	(*n =* 12) ZEA: 12/100 OTA: 12/100	ZEA: 0.12–21.87 OTA: 0.66–7.06		Immunoaffinity column	HPLC	[244]
Flour (Organic)	Hungary	(*n =* 7) 3AcDON: - 15AcDON: 2/29 D3G: - DON: - DOM: - HT-2: - T-2: - AFB1: - FB1: - OTA: - ZEA: - DAS: -	3AcDON: - 15AcDON: 62–97 D3G: - DON: - DOM: - HT-2: - T-2: - AFB1: - FB1: - OTA: - ZEA: - DAS: -		Filtration	HPLC-MS/MS	[192]
Flour (Organic)	Switzerland	(*n =* 2) ZEA: -/- ENNB: 2/100	Mean ZEA: - ENNB: 158.2		SPE	HPLC-MS/MS	[245]
Flour (Organic)	South Korea	(*n =* 15) DON: 15/100 D3G: 15/100 NIV: 15/100 15AcDON: 5/33 3AcDON: -/- Fusarenon-X: 8/53	DON: 0.74–99.86 D3G: 0.25–24.67 NIV: 0.45–79.9 15AcDON: 6.03–30.61 3AcDON: - Fusarenon-X: 1.50–4.61		Immunoaffinity column; Filtration	HPLC-MS/MS	[113]
Flour (Organic)	Croatia	(*n =* 6) AFB1: -/1 DON: 3/50 OTA: 2/33 FUM: 2/33 ZEA: 2/33	DON: 32.8–121 OTA: 2.23–2.51 FUM: 37.2–53.1 ZEA: 4.12–5.60		Filtration	ELISA	[176]
Wholemeal	Brazil	not specified	DON: 2711–3046		Immunoaffinity column; Filtration	HPLC	[168]
Wholemeal	Argentina	(*n =* 4) FB1: 3/75 FB2: 2/50 FB1 + FB2: 3/75	Mean/Max FB1: 0.28/1.10 FB2: 0.19/0.72 FB1 + FB2: 0.47/1.82		SPE	HPLC-MS/MS	[206]
Wholemeal	Turkey	(*n =* 3) AFB1: -/- AFB2: -/- AFG1: -/- OTA: 1/33	Mean AFB1: AFB2: - AFG1: - OTA: 1.06		Immunoaffinity column	HPLC-FLD	[209]
Wholemeal	Iran	(*n =* 54) AFB1: 37/69 AFB2: 35/65 AFG1: 12/22 AFG2: 12/22 Total AFs: 37/69	AFB1: 0.14–7.34 AFB2: 0.11–0.93 AFG1: 0.14–0.34 AFG2: 0.11–0.31 Total AFs: 0.44–7.61		Immunoaffinity column	HPLC-FLD	[217]
Wholemeal	China and imported (Australia, Japan, Russia, USA)	(*n =* 15) DON: 15/100	51.6–1308.9		Immunoaffinity column	HPLC-MS/MS	[220]
Wholemeal	Turkey	(*n =* 24) DON: 3/13 OTA: 8/33	DON: 1.50–2.23 OTA: 0.87–6.97		SPE; Filtration	HPLC; HPLC-FLD	[209]
Wholemeal	Portugal	(*n =* 4) D3G: 3/75 DON: 4/100 NIV: 4/100	D3G: <LOQ DON: 78.9–325.8 NIV: <LOQ-140.6		Immunoaffinity column	UPLC	[246]
Wholemeal	Hungary	(*n =* 5) 15AcDON: - 3AcDON: - D3G: - DON: 4/80 DOM: - HT-2: - T-2: - AFB1: - FB1: - OTA: - ZEA: - DAS: -	3AcDON: - 15AcDON: - D3G: - DON: 47–198 DOM: - HT-2: - T-2: - AFB1: - FB1: - OTA: - ZEA: - DAS: -		Filtration	HPLC-MS/MS	[192]
Wholemeal	UK and Germany	(*n =* 138) DON: 12/9 OTA: 22/16 T-2/HT-2: 15/11 ZEA: 20/14	Mean/St. Error DON: 63/9 OTA: 3.1/1 T-2/HT-2: 3.8/0.7 ZEA: 4.2/0.3		Filtration	LFR; ELISA	[237]
Wholemeal (Organic)	Switzerland	(*n =* 1) ZEA: -/- ENNB: 1/100	Mean ZEA: - ENNB: 97.62		SPE	HPLC-MS/MS	[245]
Wholemeal (Organic)	Hungary	(*n =* 1) 3AcDON: - 15AcDON: - D3G: - DON: - DOM: - HT-2: - T-2: - AFB1: - FB1: - OTA: - ZEA: - DAS: -	3AcDON: - 15AcDON: - D3G: - DON: - DOM: - HT-2: - T-2: - AFB1: - FB1: - OTA: - ZEA: - DAS: -		Filtration	HPLC-MS/MS	[192]
Wholemeal (Organic)	Croatia	(*n* = 12) ZEA: 12/100 OTA: 12/100	ZEA: 0.01–1.99 OTA: 0.21–4.20		Immunoaffinity column	HPLC	[244]

***Aflatoxins***—AFB1: Aflatoxin B1; AFB2: Aflatoxin B2; AFG1: Aflatoxin G1; AFG2: Aflatoxin G2; Total AFs = AFB1 + AFB2 + AFG1 + AFG2/***Alternaria Toxins***—ALP: Alterperylenol; AME: Al-ternariol monomethyl ether, AME-3-S: alternariol monomethyl ether sulfate; AOH: Alternariol; AOH-3-S: Alternariol monomethyl ether sulfate; ATX-I: Altertoxin I; TeA: Tenuazonic acid; TEN: Cyclic tetrapeptide tentoxin/***Deoxynivalenol***—15AcDON: 15-Acetyldeoxynivalenol; 3AcDON: 3-Acetyldeoxynivalenol; D3G: deoxinyvalenol-3-glucoside; DOM: de-epoxy deoxynivalenol; DON: Deoxynivalenol; Total DON: = DON + D3G + 15AcDON/***Enniatins***—ENNA: Enniatin A; ENNA1: Enniatin A1; ENNB: Enniatin B; ENNB1: Enniatin B1/***Fumonisins***—FB1: Fumonisin B1; FB2: Fumonisin B2; FB3: Fumonisin B3; HFB1: Hydrolyzed FB1; Total FUMs = FB1 + FB2 + FB3 + HFB1; FUM: FB1 + FB2 + FB3; Fumonisins: not specified/***Ochratoxins***—OTA: Ochratoxin A; OTB: Ochratoxin B/***Others***—BEA: Beauvericin; ChA: Chaetoglobosin A; CTV: Citreoviridin; DAS: Diacetoxyscirpenol; diANIV: 4,15-diacetylnivalenol; diHDAS: 7-hydroxydiacetoxyscirpenol; FUS-X: Fusarenon X; NEO: Neosolaniol; NIV: Nivalenol; STC: Sterigmatocystin/***Zearalenone***—α-ZAL: α-Ζearalanol; β-ZAL: β-Ζearalanol; β-ZOL: β-Zearalenol; α-ZOL: α-Zearalenol; Total ZEA = ZEA + α-ZOL; ZAN: Zearalanone; ZEA: Zearalenone.

### Methods of Detection of Mycotoxin in Wheat Flours

The identification of mycotoxin contamination can serve two main purposes: screening, which involves using simpler techniques and less specialized personnel, and complete quantitation, which requires expensive equipment and a high level of expertise by the analysts [245].

The analysis of mycotoxins serves a dual function: it must primarily conform to established regulations and additionally guarantee consumer safety while reducing the potential for trade rejections and the consequent economic losses [247,248]. Hence, a pivotal factor in effectively ascertaining the presence of mycotoxins is the meticulous selection of a representative sample from the entire bulk. This is accomplished through the implementation of sampling plans, which act as a method to obtain a comprehensive understanding of the presence or absence of mycotoxins. These plans are established by international authorities, such as the EU [249,250,251], USDA [252], the Codex Alimentarius [253], and the International Organization for Standardization [254].

The extraction process primarily focuses on attaining the desired compound(s) from the whole matrix while eliminating unwanted compounds that further on could hinder the analysis. Considering the fact that mycotoxins are of a hydrophobic nature except fumonisins, which are hydrophilic, their extraction is often carried out by using single or mixture of organic solvents, such as acetonitrile, chloroform, methanol, ethyl acetate [255]. The addition of buffers and/or hot water can assist solvent penetration and toxin extraction due to their ability to cleave bonds between the toxins and the sample’s constituents, such as proteins and/or sugars [256]. A commonly employed solvent mixture in the extraction of multi-mycotoxin in cereal products is a mixture of MeCN/water (84/16, *v*/*v*) [145]. It is worth noticing that while un-bound mycotoxins are easily extracted, extracting mycotoxins in their masked form is difficult and challenging. This is due to the fact that they are bound to the matrix, making the selection of the proper extraction protocol more complex [257].

**Figure 2:**
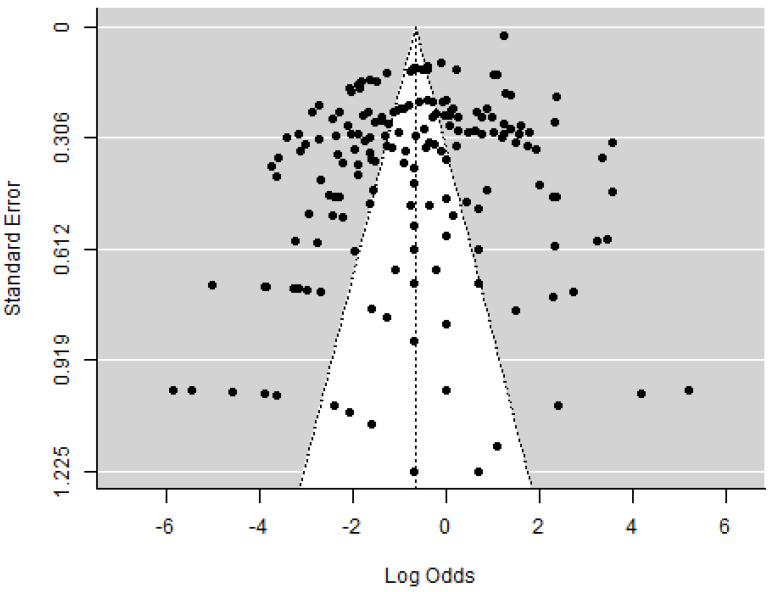
Funnel plot. Mixed Effects Model outputs.

Prior to the analysis following the extraction, a clean-up stage ensuring the removal of co-extracted compounds needs to be employed [258]. This clean-up process serves to improve the accuracy, precision, and sensitivity of the analysis. The choice of sample clean-up that will undergo subsequent high-performance liquid chromatography (HPLC) analysis depends on factors such as the sample’s nature, the analytes of interest, and the analytical technique to be used subsequently. Commonly used methods for mycotoxin extraction include filtration using a membrane or syringe filter, solid-phase extraction (SPE), or the use of immunoaffinity columns (IACs), which involve antigen-antibody interactions. The majority of the studies presented in Table 4 of this review have employed immunoaffinity columns (IACs), followed by a filtration step and solid-phase extraction (SPE) (Figure 2).

Depending on the goal of the analysis, techniques such as enzyme-linked immunosorbent assay (ELISA), lateral-flow readers (LFR), and thin-layer chromatography (TLC), are used as screening methods providing semi-quantitative results. Various analytical methods, established through interlaboratory collaborative studies conducted by international organizations and authorities, such as the Association of Official Agricultural Chemists (AOAC) International and the European Committee for Standardization (CEN), are based on high-performance liquid chromatography (HPLC) as their official methods [259]. Nevertheless, in response to the current requirements for achieving high sensitivity, specificity, and reliability, liquid chromatography coupled with tandem mass spectrometry (LC-MS/MS) has emerged as a robust technique. In the studies reviewed, white and wholemeal flour samples were predominantly analyzed using HPLC coupled with tandem MS/MS, with some instances of HPLC coupled with a fluorescent detector. Immunoassay-based methods, such as ELISA and LFR, were not extensively utilized for mycotoxin analysis.

For more comprehensive information about the extraction process, sample clean-up, and analysis, readers are encouraged to consult the reviews by Pereira et al., 2014, and Agriopoulou et al., 2020 [24,145].

## 6. Meta-Analysis

The observed log odds ranged from −5.8493 to 5.1930, with the majority of estimates being negative (63%). The estimated average log odds based on the random-effects model was µ^2^ = −0.6545 (95% CI: −0.9101 to −0.3989). Therefore, the average outcome differed significantly from zero (z = −5.0188, *p* < 0.0001). The result was back transformed from a logarithmic scale using an exponential function. Accordingly, the aggregated odds ratio turned out to be 0.52 with a lower bound confidence interval (ci.lb) value of 0.4 and an upper bound confidence interval (ci.ub) of 0.67. The upper and lower level estimated individual rate of prevalence as expressed in the odds ratio extends from a lower value of 0.01 to a higher value of 18.34.

According to the Q-test, the true outcomes appear to be heterogeneous QE (df = 193) = 4975.7015, *p* < 0.0001, τ^2^ = 2.9795, I^2^ = 97.62%). A 95% prediction interval for the true outcomes is given by −4.2182 to 2.9092. Hence, although the average outcome is estimated to be negative, in some studies, the true outcome may, in fact, be positive.

An examination of the studentized residuals revealed that none of the studies had a value larger than ±3.6735, and, hence, there was no indication of outliers in the context of this model. According to Cook’s distances, none of the studies could be considered overly influential.

A funnel plot of the estimates is shown in Figure 2. The rank correlation test indicated funnel plot asymmetry (*p* = 0.0095) but not the regression test (*p* = 0.2551).

Test of Moderators (coefficients 1:15) indicated that the model with moderators turns out to be significant with QM (df = 15) = 56.7233, pval < 0.0001, explaining part of the heterogeneity observed among studies (Table 5). The estimated effect size is not a single value. It is a value calculated for individual samples studied. In Table 5, the average of the estimated effect size is indicated in the column “estimate”. Furthermore, the upper and lower limits of the effect sizes are shown in the ci.lb (lower bound) and ci.ub (upper bound). The standard error, ‘se’, along with the statistical tests ‘pval’ and ‘zval’, determined whether the parameter is significant in the model or not.

Accordingly, a significant level of reduction in the prevalence of mycotoxin in wheat was observed in the EU and Asia categories compared to the rest. This could be explained by the strictest regulations put in place to monitor the microbiological and chemical safety of wheat traded in these regions. Being the second biggest exporter of wheat next to Russia and the second consumer next to China, the EU has put substantial effort into monitoring the safety of wheat, which has a great role in controlling the prevalence of aflatoxins. When it comes to Asia, the majority of studies included in this review representing this continent comes from China. China, being the major exporter of wheat in the region and the first consumer of wheat in the world, should respect the regulations of WTO regarding the safety of wheat and put in place its own strong policies and regulations as monitoring instruments. In the past few years, a coordinated action from China in placing stricter regulations over mycotoxin contaminants in feed and raw materials was introduced [260]. On the other hand, one of the major exporters to China’s market is the EU, which has a well-established regulatory framework on mycotoxins, leading to a relatively smaller degree of mycotoxin presence, as stated in the previous sections, compared to other geographical areas. This would allow China to receive wheat from less mycotoxin-prevalent regions. All these are of paramount importance in reducing the prevalence of aflatoxins in wheat and other commodities derived from wheat.

Among the flour types, those prepared for infants showed a significant reduction as compared to the other commercial wheat flours. This is also related to the close monitoring of inputs destined for the manufacturing of infant formula. Many countries have put in place stronger regulations along the value chain of inputs for infant formula compared to conventional wheat flour. This would definitely contribute to the reduction of mycotoxins in infant formula.

Among the major family of the grouped mycotoxin families, the prevalence level has shown a significant increase with Deoxynivalenol types. Again, this could be explained by the fact that DON attracts the highest interest in the literature, being the most extensively studied mycotoxin. The major findings from the meta-analysis highlight the importance of putting in place policy instruments to closely monitor production, storage, and processing, and handle practices of wheat along its value chain to minimize the food safety risks arising from the exposure of mycotoxins.

## 7. Conclusions

Cereal grains and products derived from them, such as flour, hold a significant role in global nutrition. Consequently, they exert a substantial influence on human exposure to mycotoxins. International regulatory bodies are actively involved in closely monitoring mycotoxin issues across the food chain, but these efforts vary across different geographical regions, and in some areas, they are entirely lacking. This underscores the necessity for collective action, emphasizing the importance of knowledge exchange among diverse regulatory authorities to create universally effective measures that facilitate global transportation and trade.

To date, the most effective strategies for preventing mycotoxin formation involve implementing Good Agricultural Practices (GAPs) and Hazard Analysis and Critical Control Points (HACCP) at both pre- and post-harvest stages. These systems enable comprehensive management to minimize the presence of highly toxigenic fungal genera, such as *Fusarium*, *Aspergillus*, and *Penicillium*. Since filamentous fungi can produce multiple mycotoxins, the presence of a specific mycotoxin may serve as an indicator for others. However, targeted analyses often fail to identify potentially coexisting mycotoxins, leading to possible underestimations. This raises the possibility that supposedly “emerging mycotoxins” may actually be already existent rather than truly “emerging”.

Fungal and mycotoxin contaminants tend to be more concentrated in the bran of the kernel rather than in the inner fractions of the grain. However, the excessive contamination levels observed in the white flour samples did not significantly differ from those in the whole-milled ones. It is worth noting that the traditional identification of microorganisms and the analytical methods employed for mycotoxin quantification have inherent limitations that can potentially impact the results. The studies presented laid the greatest emphasis on quantifying DON, with nearly double the focus compared to aflatoxins, zearalenone, and OTA. DON exhibited the highest prevalence, with lower contamination levels observed across the European Union (EU) and China. In light of the points mentioned earlier, the importance of vigilant and ongoing monitoring of fungal and mycotoxin contaminants becomes increasingly crucial, especially during this period of global social and economic instability, which places strain on the sourcing of cereals. Professionals involved in this field should take proactive measures to mitigate the immediate consequences of these issues, which can lead to food scarcity, hunger, malnutrition, and associated health risks. Such efforts are essential for raising consumer awareness and addressing these pressing challenges.

## Figures and Tables

**Table 2:** Occurrence of Fungal Contaminants in Flour.

Flour Origin and Type	Location	Number of Samples Tested	Fungal Species ^1^ (Frequency, %)	Fungal Load (log CFU/g unless Stated Differently) ^2^	Detection Techniques Applied	References
Wheat Flour	Iraq	n = 3	*Aspergillus* spp. (21.3%)—*A. flavus*, *A. niger*, *A. orchraceus* *Penicillium* spp. (15.84%), *Fusarium* spp. (12.23%) Lower Frequency—*Rhizopus* spp., *Ulocladium* spp.	3.0–4.5	Direct Plating Techniques (PDA)	[77]
Wheat Flour	Iran	n = 80	*Penicillium* spp. (24.29%), *Cladosporium* spp. (20%), *Mucor* spp. (20%), *Aspergillus* spp. (19.29%), *Alternaria* spp. (3.57%), *Rhizopus* spp. (2.14%)	3.8	Dilution Plate Method (PCA, YGCA, MEA) and DNA-Based Techniques	[10]
Wheat Flour	Australia	n = 81	*Aureobasidium* spp., *Cladosporium* spp., *Alternaria* spp., *Fusarium* spp., *Penicillium* spp., *Aspergillus* spp., *Eurotium* spp., *Rhizopus* spp., *Mucor* spp.	Not specified	Direct Plating Techniques (DRBC + DG-18)	[78]
Wheat Flour	Spain	n = 26	*Aspergillus* spp. (*A. candidus*, *A. flavus*, *A. fumigatus*, *A. ochraceus*, *A. versicolor*, *A. rubrum*, *A. niger*), *Penicillium verrucosum*	Not specified	Direct Plating Techniques (DRBC + MEA + CYA + G25N)	[79]
Wheat Flour (Self-raising)	USA	n = 5	*Alternaria* spp., *Aspergillus* spp., *Cladosporium* spp., *Eurotium* spp., *Fusarium* spp.	2.0–3.0	Direct Plating Techniques (PDA + DG-18) and DNA-Based Techniques for Identification	[71]
Wheat Flour (unbleached)	USA	n = 12	*Alternaria* spp., *Aspergillus* spp. (*A. flavus*), *Fusarium* spp. (F. *graminearum*), *Penicillium* spp.	2.0–3.0	Direct Plating Techniques (PDA + DG-18) and DNA-Based Techniques for Identification	[71]
Whole Wheat Flour	USA	n = 5	*Alternaria* spp., *Aspergillus* spp. (A. flavus), *Fusarium* spp., *Penicillium* spp., *Cladosporium* spp.	2.0–3.0	Direct Plating Techniques (PDA + DG-18) and DNA-Based Techniques for Identification	[71]
Wheat Flour, Barley Flour, Cake Flour	Iran	n = 179	*Aspergillusfumigatus*, *Aspergillusniger*	2.0–3.0	Direct Plating Techniques (ADRBC) and DNA-Based Techniques for Identification	[80]
Whole Wheat Flour	Brazil	n = 50	*Penicillium* spp. (38.2%), *Aspergillus* spp. (23.6%), Aspergillus spp., *Eurotium*-*typeascomata* (19.1%)	3.1	Direct Plating Techniques (DG 18 and DRBC)	[63]
Whole Corn Flour	Brazil	n = 5	*Penicillium polonicum* (42.9%) and *Fusarium* spp. (28.6%)	4.8	Direct Plating Techniques (DG 18 and DRBC)	[63]
White Wheat Flour	Serbia	Not specified	*A. versicolor*, *C. cladosporioides*, *F. sporotrichoioides*, *P. aurantiogriseum*, *P. expansum*	DG18 −1.78 MY50G −1.47	Direct Plating Techniques (DG 18 and MY50G, CYA)	[81]
Whole Wheat Flour	Serbia	Not specified	*C. cladosporioides*, *F. proliferatum*, *P. expansum*	DG18 −2.11 MY50G −1.60	Direct Plating Techniques (DG 18 and MY50G, CYA)	[81]
Corn Flour	Serbia	Not specified	*A. flavus*, *A. niger*, *F. sporotrichoioides*, *F. proliferatum*, *P. commune*, *P. oxalicum*, *Rhizopus stolonifer*	DG18 −2.53 MY50G −2.43	Direct Plating Techniques (DG 18 and MY50G, CYA)	[81]
Whole Buckwheat Flour	Serbia	Not specified	*Alternaria alternata*, *A. fumigatus*, *C. cladosporioides*, *Chrysonilia sitophila*, *P. aurantiogriseum*	DG18 −2.70 MY50G −1.47	Direct Plating Techniques (DG 18 and MY50G, CYA)	[81]
Wheat Flour	Egypt	n = 29	*Aspergillus* spp. (58.2%) (*A. flavus* (27.8%), *A. niger* (14.6%), *A. parasiticus* (7.2%), *Penicillium* spp. (15.2%), *Mucor circinelloids* (7.2%)	6.7–1356.9 (ATC/g)	Dilution Plate Method (CZA)	[82]
Wheat Flour Type “00”	Italy	n = 3	*Alternaria* spp. –*Alterniainfectoria*, *Aspergillus* spp., *Aspergillus fasiculatus*, *A. oryzae*, *A. clavatus*, *Chaetomium globosum.*, *Cladosporium* sp., *Epicoccum nigrum*, *Fusarium oxysporum*, *Mucor* sp., *Penicillium aurantiogriseum*, *Penicillium* sp., *Penicillium albocoremium*, *Penicillium chrysogenum*, *Rhizopus oryzae*, *Penicillium citrinum*	Not specified	Direct Plating Techniques (PDA) and ITS-amplicon metabarcoding analysis	[39]
Wheat Flour “00”	Italy	n = 3	*Alternaria* spp., *P. griseofulvum*, *P. verrucosum*, *P. aurantiogriseum*, *P. viridicatum*, *P. polonicum*, *Penicillium* sp., *Cladosporium* sp., *Arthriniumarundinis*	Not specified	Direct Plating Techniques (PDA) and ITS-amplicon metabarcoding analysis	[39]
Whole Wheat Flour	Italy	n = 3	*Alternaria* sp., *Penicillium* sp., *Penicillium aurantiogriseum*, *Penicillium allii*-*sativi*, *P. chrysogenum*, *Penicillium griseofulvum*	Not specified	Direct Plating Techniques (PDA) and ITS-amplicon metabarcoding analysis	[39]
Wheat Flour	Saudi Arabia	n = 50	*Aspergillus* spp. (70%), *Penicillium* spp. (30%), *Eurotium* spp. (14%), *Fusarium oxysporum* (20%), and *Alternaria* *alternata* (18%)	224, 116, 109, 75, 64 (ATC/g)	Direct Plating Techniques (MEA and DRBC)	[83]
Various Flours (Mainly wheat and corn)	Italy	n = 40	*Aspergillus* spp., *Fusarium* spp., *Penicillium* spp.	Not specified	Plating Dilution Technique (SDA and DG18) and ITS DNA Phylogenetic studies	[84]
Wheat Flour	Egypt	n = 30	*Aspergillus flavus*, *A. nigri*, *Penicillium* spp.	2.91	Dilution Plate Method (CMA, YES, ADM)	[85]
Wheat flour	Egypt	n = 20	*Aspergillusflavus*, *A. niger*, *A. versicolor*, *Penicilliumducluxi*, and *Rhizophusnigricans*	0.34–1.45	Dilution Plate Method (CZA)	[58]
Wheat Flour	Nigeria	n = 3	*A. flavus* (33.0%), *A. niger* (11.0%), *Rhizopus* spp. (11.0%), *Paecilomyces* spp. (11.0%), *Yeast* spp. (11.0%), and *Geotrichum* spp. (11.0%)	12–13.5	Dilution Plate Method (PDA)	[86]
Wheat Flour	Iran	n = 89	*Aspergillus* spp. (50%) (*A*. *niger* and *A. fumigatus*), *Fusarium* spp. (18.0%), *Acremonium* spp. (14.5%), *Mucor* spp. (7.0%), *Penicillium* spp. (3.5%)	>4	Dilution Plate Method (YCGA)	[87]

^1^ Main fungal contaminants reported and frequency data reported in %. ^2^ Quantitative data reported in variable units as reported by various research studies.

**Table 5:** Model outputs for Mixed Effects model with fifteen moderators.

	Estimate	se	zval	pval	ci.lb	ci.ub	
**Area**							
Africa	−0.7411	0.7208	−1.0282	0.3039	−2.1538	0.6716	
Asia	−0.7974	0.3374	−2.3631	0.0181	−1.4588	−0.136	**
Australia	1.0529	1.8022	0.5843	0.5590	−2.4792	4.5851	
EU	−1.2728	0.4537	−2.8054	0.0050	−2.1620	−0.3836	***
MERCOSUR	−0.6387	0.5648	−1.1308	0.2581	−1.7456	0.4683	
**Mycotoxin type**							
*Alternaria* mycotoxins	0.3667	0.5702	0.6431	0.5201	−0.7509	1.4843	
Deoxynivalenol	0.7081	0.4022	1.7607	0.0783	−0.0801	1.4963	*
Enniatins	0.4083	0.5079	0.8040	0.4214	−0.5871	1.4037	
Fumonisins	0.8386	0.6337	1.3233	0.1857	−0.4035	2.0806	
Ochratoxins	−0.0667	0.5786	−0.1153	0.9082	−1.2007	1.0672	
Others	−0.1656	0.5268	−0.3143	0.7533	−1.1982	0.8670	
T-2 and HT-2	−1.0085	0.7683	−1.3126	0.1893	−2.5143	0.4974	
Zearalenone	−0.8837	0.5519	−1.6012	0.1093	−1.9655	0.1980	
**Flour Type**							
Flour (Infants)	−1.5589	0.7859	−1.9836	0.0473	−3.0992	−0.0186	**
Flour (whole and plain)	0.1627	0.4506	0.3611	0.718	−0.7205	1.0459	

Significance levels: 0.001 ‘***’; 0.01 ‘**’; 0.05 ‘*’.
